# eIF4E phosphorylation recruits β-catenin to mRNA cap and promotes Wnt pathway translation in dentate gyrus LTP maintenance

**DOI:** 10.1016/j.isci.2023.106649

**Published:** 2023-04-15

**Authors:** Sudarshan Patil, Kleanthi Chalkiadaki, Tadiwos F. Mergiya, Konstanze Krimbacher, Inês S. Amorim, Shreeram Akerkar, Christos G. Gkogkas, Clive R. Bramham

**Affiliations:** 1 Department of Biomedicine Jonas Lies vei 91, University of Bergen, 5009 Bergen, Norway; 2 Biomedical Research Institute, Foundation for Research and Technology-Hellas, 45110 Ioannina, Greece; 3 Mohn Research Center for the Brain, University of Bergen, Bergen, Norway; 4 Center for Discovery Brain Sciences, University of Edinburgh, EH8 9XD Edinburgh, UK

**Keywords:** Molecular biology, Cell biology

## Abstract

The mRNA cap-binding protein, eukaryotic initiation factor 4E (eIF4E), is crucial for translation and regulated by Ser209 phosphorylation. However, the biochemical and physiological role of eIF4E phosphorylation in translational control of long-term synaptic plasticity is unknown. We demonstrate that phospho-ablated *Eif4e*^*S209A*^ Knockin mice are profoundly impaired in dentate gyrus LTP maintenance *in vivo*, whereas basal perforant path-evoked transmission and LTP induction are intact. mRNA cap-pulldown assays show that phosphorylation is required for synaptic activity-induced removal of translational repressors from eIF4E, allowing initiation complex formation. Using ribosome profiling, we identified selective, phospho-eIF4E-dependent translation of the Wnt signaling pathway in LTP. Surprisingly, the canonical Wnt effector, β-catenin, was massively recruited to the eIF4E cap complex following LTP induction in wild-type, but not *Eif4e*^*S209A*^, mice. These results demonstrate a critical role for activity-evoked eIF4E phosphorylation in dentate gyrus LTP maintenance, remodeling of the mRNA cap-binding complex, and specific translation of the Wnt pathway.

## Highlights

• Synaptic activity-induced eIF4E phosphorylation controls DG-LTP maintenance *in vivo*
• eIF4E phosphorylation triggers release of translational repressors from cap complex
• eIF4E phosphorylation recruits β-catenin to cap complex
• eIF4E phosphorylation selectively enhances translation of Wnt pathway

## Graphical abstract

**Figure:**
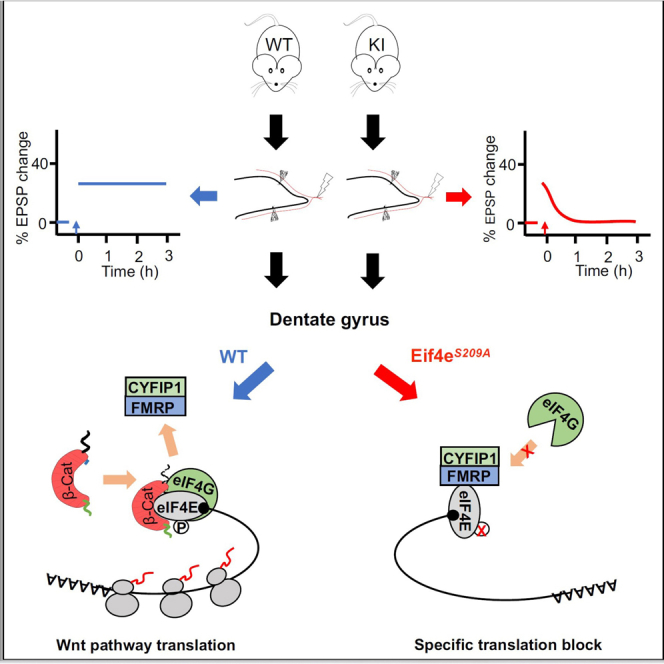

## Introduction

Neuronal activity-dependent synaptic plasticity is crucial for adaptive behaviors such as memory formation,^1^ and dysregulation of plasticity is commonly found in animal models of neurodevelopmental and degenerative disorders. At excitatory glutamatergic synapses, the generation of stable structural and functional changes lasting hours or more requires *de novo* gene expression and protein synthesis.^2^^,^^3^^,^^4^ Global gene expression profiles in various plasticity models have been elucidated using microarrays and RNA-sequencing^5^^,^^6^^,^^7^^,^^8^^,^^9^ as well as ribosome profiling.^10^^,^^11^ In neurons, regulation of gene expression at the level of translation is critical for long-term synaptic plasticity.^12^^,^^13^

Translation initiation, the multistep process by which the ribosome is recruited to mRNA, is tightly regulated and often rate-limiting for protein synthesis.^14^ Eukaryotic translation initiation factor 4E (eIF4E), which binds to the 5′-terminal cap structure of cytoplasmic mRNA, plays a key role in both the process and regulation of translation. eIF4E enables assembly of a multiprotein translation initiation complex at the mRNA 5′ end, which recruits the 40S small ribosomal subunit and scans to the mRNA start codon. Interaction of eIF4E with the scaffolding protein eIF4G is crucial for initiation complex formation. This interaction is obstructed by eIF4E-binding proteins (4E-BPs). Hypophosphorylated 4E-BPs bind to eIF4E, competes for eIF4G binding and represses translation initiation. Signaling to the kinase mechanistic target of rapamycin (mTORC1) enhances translation by phosphorylation of 4E-BPs, leading to their dissociation from eIF4E.^15^^,^^16^ In a major convergent pathway, activation of extracellular signal-regulated kinase (ERK, aka mitogen-activated protein kinase; MAPK) signaling to MAPK-interacting kinases (MNK1 and MNK2) phosphorylates eIF4E on a single residue, Ser209.^17^^,^^18^ Phosphorylation of eIF4E is usually, but not always, associated with enhanced translation initiation.^19^^,^^20^^,^^21^^,^^22^ Thus, the molecular function of Ser209 eIF4E phosphorylation is unresolved.^23^

Translational control in synaptic plasticity has been extensively studied in excitatory pathways of the hippocampus. A major question is whether translation mechanisms are differentially implemented to sculpt protein synthesis and plasticity in a pathway-specific manner. In the hippocampal CA1 region, ERK-dependent translation initiation regulates stable LTP formation.^24^^,^^25^ Surprisingly, eIF4E phosphorylation is dispensable at Schaffer collateral-CA1 synapses, as LTP induction and maintenance are normal in knockin mice harboring nonphosphorylatable Ser209Ala mutated eIF4E (*Eif4e*^*S209A*^ or *Eif4e*^*ki*^).^26^ At the medial perforant path input to the dentate gyrus (DG), studies employing kinase inhibitors and MNK knockout mice implicate ERK-MNK signaling in enhanced translation initiation and LTP maintenance.^27^^,^^28^ However, the specific role of eIF4E phosphorylation in DG-LTP maintenance and translation control has not been investigated.

Here, we used *Eif4e*^*ki*^ mice to explore the role of eIF4E phosphorylation in DG-LTP *in vivo*. We demonstrate that ablation of Ser209 phosphorylation inhibits the synaptic activity-evoked discharge of eIF4E repressor proteins, prevents formation of the translation initiation complex, and selectively inhibits LTP maintenance without affecting basal synaptic transmission or LTP induction. Using unbiased ribosome profiling, we show that eIF4E phosphorylation is required for specific translation of Wnt pathway targets during DG-LTP. In canonical Wnt signaling, β-catenin mediates transcriptional regulation.^29^^,^^30^ Here we report a previously unknown, massive recruitment of β-catenin to eIF4E following LTP induction. The discharge of repressors and recruitment of β-catenin both depend on synaptic activity-evoked phosphorylation of eIF4E. Collectively, these results demonstrate a major function for Ser209 phosphorylation in stimulus-induced remodeling of eIF4E interactions and enhanced Wnt pathway translation in LTP maintenance.

## Results

### Loss of eIF4E Ser209 phosphorylation selectively inhibits DG-LTP maintenance

We first examined the impact of ablating phospho-eIF4E on basal perforant path-DG transmission in adult anesthetized mice. Evoked field potentials were obtained across a range of stimulation intensities and input-output curves were constructed of the field EPSP slope (Figure S1A), population spike amplitude (Figure S1B), and plots of the EPSP-population spike relationship were made to evaluate synaptic excitability of granule cells (Figure S1C). Homozygous *Eif4e*^ki/ki^ mice were not significantly different from wild-type in any of these measures, indicating that ablation of phospho-eIF4E does not affect synaptic efficacy or granule cell excitability.

**Figure 1:**
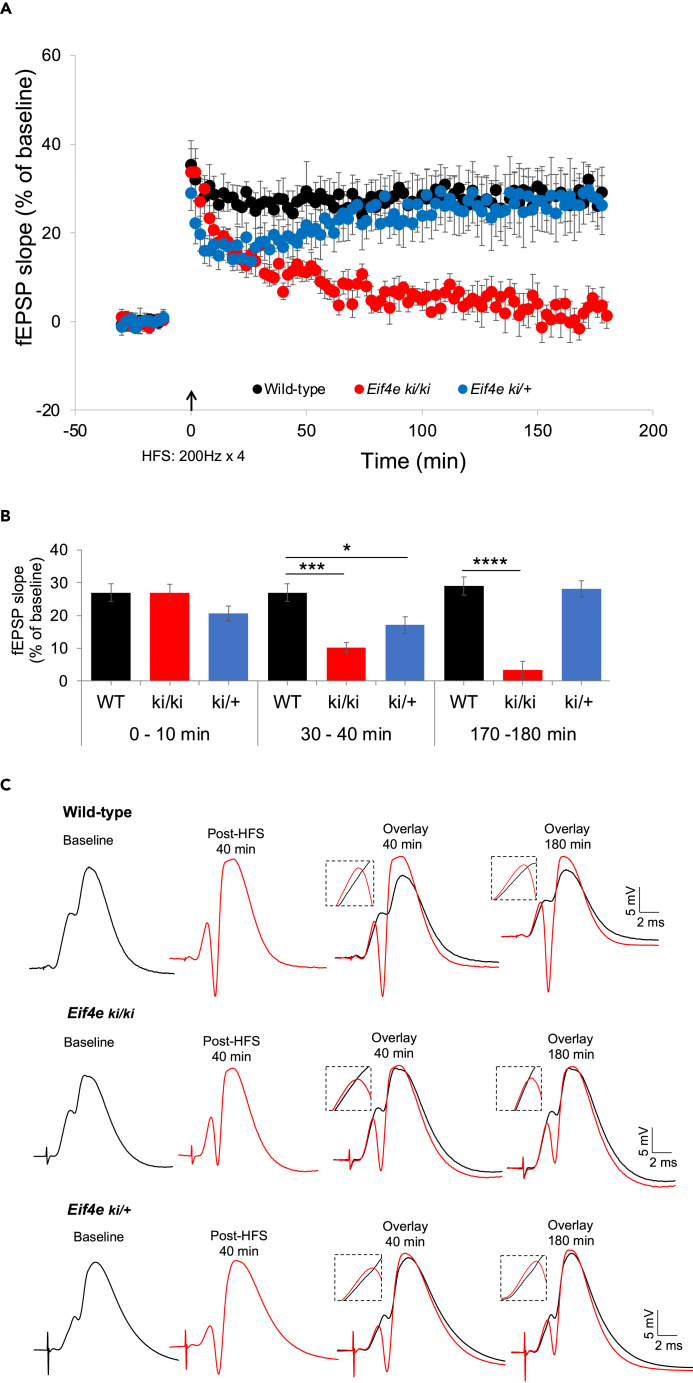
Ablation of phospho-eIF4E selectively impairs DG-LTP maintenance *in vivo* (A) Time-course plots of medial perforant path-dentate gyrus (DG) evoked fEPSPs recorded before and after high-frequency stimulation (HFS, indicated by arrow) in homozygous *Eif4e*^*S209A*^ knockin mice (*Eif4e*^ki/ki^; n = 7), heterozygous mice (*Eif4e*^+/ki^; n = 8) and wild-type littermates (*Eif4e*^+/+^ mice; n = 10). Values are mean ± SEM of the maximum fEPSP slope expressed in percent of baseline. (B) Bar graphs show mean changes in fEPSP slope recorded between 0 and 10 min, 30–40 min, and 170–180 min post-HFS. Significant differences between genotypes: ∗p < 0.05; ∗∗∗p < 0.0001, ∗∗∗∗p < 0.00001; Student’s *t* test. Heterozygotes exhibit transient impairment in early LTP maintenance. Homozygous show loss of stable LTP maintenance. (C) Representative field potentials recorded at baseline and post-HFS (40 min and 180 min). Each trace is the average of 4 consecutive responses. See also Figures S1 and S2.

We then asked whether ablation of eIF4E phosphorylation impacts LTP induced by application of high-frequency stimulation (200 Hz, 4 trains of 15 pulses) (Figure 1). In wild-type mice, HFS induced an increase in fEPSP slope which remained stable during 3 h of post-HFS recording (Figure 1A). In *Eif4e*^ki/ki^ mice, HFS induced an initial increase in fEPSP slope that was not significantly different in magnitude from wild-type control at 0–10 min post-HFS (Figures 1A and 1B). However, in striking contrast to wild-type, the fEPSP increase declined completely to baseline by 2 h post-HFS. Already at 30–40 min post-HFS, *Eif4e*^ki/ki^ mice exhibited a severe (60.6%) reduction in fEPSP potentiation relative to wild-type (Figures 1B and 1C). Like homozygotes, heterozygous *Eif4e*^ki/+^ mice showed impaired LTP at 30–40 min post-HFS (Figures 1B and 1C). However, the inhibition in heterozygotes was transient, as fEPSP responses returned to the wild-type control level to exhibit stable LTP (Figures 1A and 1B). The results show that phosphorylation of eIF4E is required for maintenance of synaptic LTP, but dispensable for basal transmission and LTP induction. Of interest, *Eif4e*^ki/ki^ mice showed a stable increase in the population spike, indicating that mechanisms other than eIF4E phosphorylation regulate plasticity of granule cell excitability (Figures 1C, S2A, and S2B).

### Loss of eIF4E phosphorylation inhibits stimulus-induced release of eIF4E repressors and prevents initiation complex formation in DG-LTP *in vivo*

**Figure 2:**
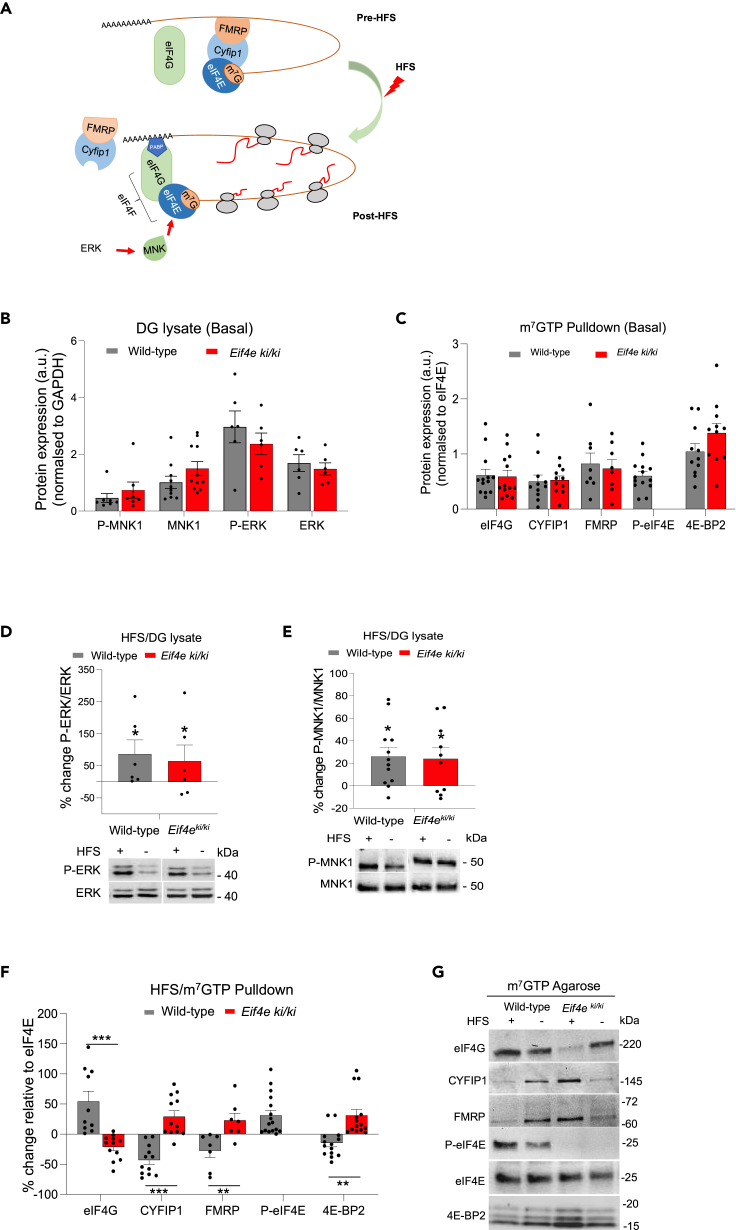
Ablation of phospho-eIF4E impairs synaptic activity-evoked discharge of eIF4E repressors and assembly of translation initiation complex (A) Model of HFS-evoked, MNK1-catalyzed eIF4E phosphorylation resulting in discharge of the CYFIP1/FMRP (Fragile-x messenger ribonucleoprotein) complex from eIF4E and recruitment of eIF4G to form the translation initiation complex. Based on Panja et al. (2014).^28^ (B) Immunoblot analysis of total (T) and phosphorylated (P) ERK and MNK in DG lysates from naive mice (basal state). No significant difference between wild-type and *Eif4e*^ki/ki^ mice in expression of active, P-ERK (Thr202/Tyr204), P-MNK (Thr197/202), or expression of their respective total proteins normalized to GAPDH. (P-ERK, n = 6; ERK, n = 6; P-MNK1, n = 7; MNK1, n = 11) Values are means +SEM. (C) Immunoblot analysis from m^7^GTP pulldown assays in DG lysates in naive mice (basal state). Densitometric values for eIF4E cap-associated proteins (eIF4G, 4E-BP2, CYFIP1 and FMRP) are normalized to values of m^7^GTP-bound eIF4E. Ablation of phospho-eIF4E in *Eif4e*^ki/ki^ mice did not alter translation factor binding relative to wild-type. (D) (Top). Enhanced ERK phosphorylation in ipsilateral, HFS-treated DG. Values in lysates sample are expressed in percent change relative to contralateral DG control. P-ERK is normalized to total ERK. Upper and lower bands (ERK1/ERK2) combined for quantification. (Bottom). Representative immunoblots. ∗p < 0.05; Student’s *t* test. (E) (Top). Enhanced MNK1 phosphorylation in ipsilateral, HFS-treated DG. Values in lysates sample are expressed in percent change relative to contralateral DG control. P-MNK1 is normalized to total MNK1. (Bottom). Representative immunoblots. HFS = high-frequency stimulation. (+) Ipsilateral DG, (−) Contralateral DG. In panels D and E, ∗ indicates significant increase in HFS-treated DG relative to contralateral control. ∗p < 0.05; Student’s *t* test. (F) Immunoblot analysis of m^7^GTP pulldowns from DG lysates obtained 40 min post-HFS. Values normalized to levels of m^7^GTP-bound eIF4E and expressed in percent changes in HFS-treated DG relative to contralateral control. eIF4G = 0.00017, CYFIP1= 0.00004, FMRP= 0.008, T-BP2= 0.0005 (∗∗p < 0.001, ∗∗∗p < 0.0001; Multiple t-test). (G) Representative immunoblots for panel F. See also Figure S3.

Next, we examined the biochemical function of eIF4E phosphorylation. Previous work showed that pharmacological inhibition of MNK prevents eIF4E phosphorylation and formation of the translation initiation complex during DG-LTP. According to this model, MNK activity triggers discharge of eIF4E repressors: cytoplasmic FMRP interacting protein-1 (CYFIP1) and the canonical eIF4E-binding protein (4E-BP2)^27^^,^^28^ (Figure 2A). However, the specific role of eIF4E phosphorylation is unknown, as MNK has multiple additional substrates with roles in translation and mRNA metabolism.^19^^,^^20^^,^^31^

First, we asked whether ablation of Ser209 eIF4E phosphorylation impacts basal ERK-MNK signaling and initiation complex formation. In unstimulated DG tissue from naive mice, expression of total and phosphorylated (activated) ERK and MNK did not significantly differ between *Eif4e*^ki/ki^ mice and wild-type (Figure S2B). eIF4E binds to the 7-methylguanosine (m^7^GTP) moiety of the 5′-terminal mRNA cap. To assess regulation of the eIF4E cap-binding complex, m^7^GTP cap-pulldowns were performed in DG lysates and immunoblotting was used to quantify levels of interacting proteins relative to eIF4E. Phospho-eIF4E was present in cap-pulldown samples from wild-type mice, but absent from *Eif4e*^ki/ki^ mice. However, there was no difference between genotypes in eIF4G binding to eIF4E, indicating normal formation of the eIF4F initiation complex (Figure 2C). Levels of cap/eIF4E-associated CYFIP1, FMRP, and 4E-BP2 in *Eif4e*^ki/ki^ mice were also not different from wild-type (Figure 2C). Immunoblotting of DG lysate (input) samples similarly showed no genotype differences in expression of the translation factors relative to GAPDH loading control (Figure S3A). Thus, under basal conditions, absence of eIF4E phosphorylation did not alter ERK-MNK signaling or eIF4E protein-protein interactions.

Next, we analyzed ERK-MNK signaling in response to perforant path stimulation. At 40 min post-HFS, expression of phospho-ERK and phospho-MNK was significantly enhanced in ipsilateral DG relative to the contralateral, non-stimulated DG, with no significant difference between genotypes (Figures 2D and 2E). Thus, HFS-induced ERK-MNK signaling is intact and unaltered in *Eif4e*^ki/ki^ mice. We then performed cap-pulldown assays in DG lysates to assess changes in eIF4E interactions. In wild-type mice, binding of CYFIP1, FMRP, and 4E-BP2 to eIF4E was significantly reduced while loading of eIF4G was enhanced, relative to the contralateral control DG (Figures 2F and 2G). In contrast, HFS in *Eif4e*^ki/ki^ mice failed to discharge the repressor proteins or enhance eIF4G binding to eIF4E (Figures 2F and 2G). Rather, we observed increased recovery of CYFIP1 and 4E-BP2 in the cap-pulldown in *Eif4e*^ki/ki^ mice, suggesting stabilization of the interaction complex in the absence of eIF4E phosphorylation. In DG lysates, CYFIP1 levels decreased in HFS-treated wild-type mice and increased in *Eif4e*^ki/ki^ mice, consistent with degradation of CYFIP1 after release from eIF4E (Figures S3B and S3C). Taken together, these results suggest that stimulus-evoked phosphorylation of eIF4E on Ser209 is required for the release of translational repressors, formation of the translation initiation complex and maintenance of LTP.

Translation of the immediate-early gene Arc is causally implicated in DG-LTP consolidation,^32^ and regulated by MNK signaling in rats and mice.^27^^,^^33^ In the present study, HFS-induced Arc expression was significantly reduced in *Eif4e*^ki/ki^ mice relative to wild-type (Figures S3B and S3C), whereas basal Arc expression did not differ between genotypes (Figure S3A). Taken together these data suggest that Arc expression in LTP specifically depends on MNK catalyzed phosphorylation of eIF4E.

### Ribosome profiling identifies phospho-eIF4E-dependent specific translation in LTP: Enhanced translation of Wnt signaling pathway

**Figure 3:**
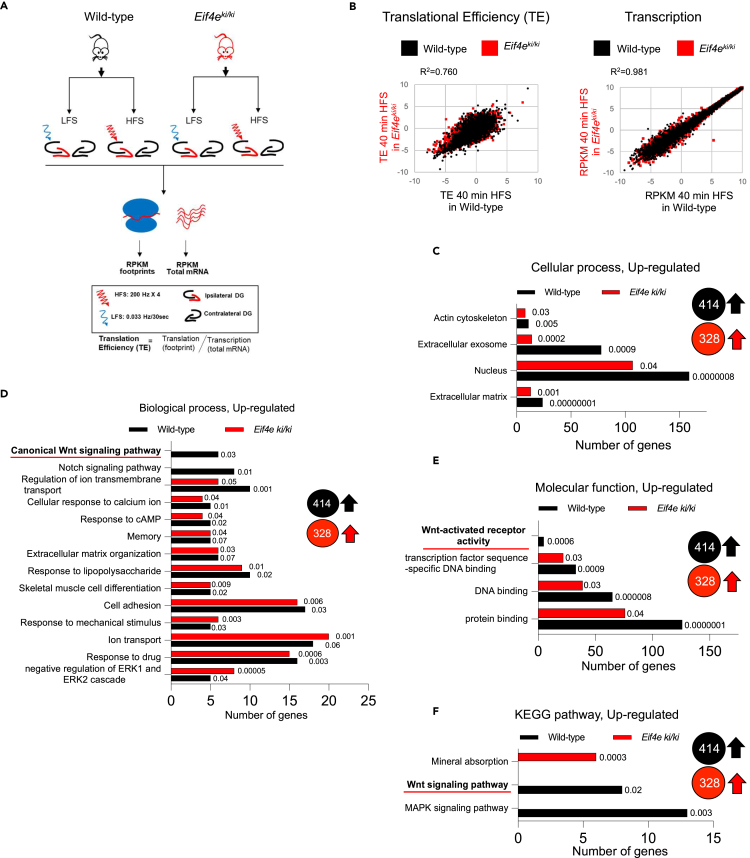
Ribosomal profiling identifies phospho-eIF4E-dependent specific translation of Wnt family pathway in DG-LTP *in vivo* (A) Schematic representation of design for ribosomal profiling experiments. (B–F) (B) Scatterplot of log2 TE Plot showing upregulated translational efficiency and transcription in *Eif4e*^ki/ki^ mice versus *Eif4e*^+/+^ (wild-type) libraries following HFS stimulation for 40 min (p<0.05 and 0.75≥ TE ratio≤1.5). Gene ontology analysis of upregulated genes in 40 min HFS *Eif4e*^+/+^ (414) and *Eif4e*^ki/ki^ mice (328); plots for cellular component (C), biological process (D) and molecular function (E) with number of genes in each category with pvalues. (F) KEGG pathway analysis for upregulated genes. Mean ± SEM and Student’s *t* test in Table S1. LFS: low-frequency test pulse stimulation only, HFS: high-frequency stimulation, DG: Dentate Gyrus. See also Figures S5 and S6.

Next, we used unbiased ribosome profiling to identify changes in translational activity linked to phospho-eIF4E-dependent maintenance of LTP. This analysis focused on 40 min post-HFS as a critical time in the transition to stable LTP. In the LTP experiments, mice received HFS and standard low-frequency test-pulse stimulation (LFS) to assess changes in the fEPSP. The ipsilateral, HFS-treated DG and the contralateral unstimulated DG were collected at 40 min post-HFS) (Figure 3A). To control for effects of test-pulse stimulation, a control group received LFS only (Figure 3A). To ascertain the effect of HFS, we normalized the data from ipsilateral HFS-treated DG to LFS-treated DG from respective WT and *Eif4e*^ki/ki^ mice. We prepared RNA sequencing libraries from both ribosome-protected footprints (a proxy for translation) and total mRNA (a proxy for transcription) (Figure 3A). Novaseq produced high quality reads for footprints and mRNA libraries because: (1) the distribution of footprint size (28-32 nt) is canonical (Figure S4B, top panel), (2) the read distribution within the three frames is more abundant for the protein coding frame (Figure S4B, bottom panel), and (3) the periodicity of ribosomal footprints across mRNA coding and non-coding regions is canonical (Figure S4C).

First, we aimed to elucidate the translational landscape of LTP in wild-type (*Eif4e*^+/+^) mice 40-min after HFS. In terms of global mRNA translation (TE was calculated by the RPKM reads of footprints normalized to mRNA abundance) and global mRNA abundance, there was no significant effect of HFS treatment relative to contralateral DG or LFS-treated DG (Figure S5; Table S2). However, we detected an overall increase in mRNA-specific translation at 40 min post-HFS of differentially translated genes (DTGs) and an overall increase in transcription of differentially expressed genes (DEGs), including known immediate-early genes (IEGs) such as Arc, Junb, Npas4, Fos and Fosb (Figure S5 and Table S2). Gene ontology analysis of DTGs and DEGs using DAVID, and Ingenuity pathway analysis (IPA) revealed LTP-related categories, such as calcium, post-synapse, excitatory post-synapse potential and key biological pathways, such as AMPK signaling, actin cytoskeleton and extracellular matrix (Figure S5 and Table S3).

Second, we investigated the effect of the ablation of Ser209 phosphorylation, focusing on translational efficiency (TE) at 40 min post-HFS. TE was calculated by the RPKM reads of footprints normalized to mRNA abundance. Although we did not detect significant changes in global mRNA levels between HFS-treated wild-type and *Eif4e*^ki/ki^ (Figure 3B, R^2^ = 0.981), there was a modest upregulation of translation (Figure 3B, R^2^ = 0.760). Consequently, analysis of log_2_ of TE between wild-type and *Eif4e*^ki/ki^ mice at 40 min post-HFS, as compared to LFS-treated control (ratio<0.667&ratio>1.5; p<0.05), identified that 471 genes in wild-type mice (414 upregulated and 57 downregulated) and 419 genes in *Eif4e*^ki/ki^ mice (328 upregulated and 91 downregulated) were differentially translated (Table S4). We then performed GO analysis using the DAVID (database for annotation, visualization, and integrated discovery) platform^34^ (Figure 3 and Table S3). Key GO categories identified for upregulated DTGs both in wild-type and *Eif4e*^ki/ki^ mice include memory, extracellular matrix organization, cell adhesion, cellular response to calcium ion and actin cytoskeleton (Figures 3C and 3D, Table S3). Strikingly, the canonical Wnt signaling pathway and its related genes were absent from the *Eif4e*^ki/ki^ mice GO analysis, whereas they were significantly upregulated in wild-type mice (Figures 3D–3F, Table S3). Within this GO Wnt signaling pathway category, we identified translationally upregulated Wnt4, Wnt receptors (Lrp5, Fzd2, Fzd4), and the receptor scaffolding protein disheveled 2 (Dvl2) (Table S5). In addition, major β-catenin transcriptional targets (Adamts10, Bcl2l2, Ppard, Vegf, Hspg2, pkd1) exhibited enhanced translational efficiency at 40 min post-HFS relative to control mice given LFS only (Table S5). Key GO categories among downregulated DTGs in *Eif4e*^ki/ki^ mice, as compared to wild-type, include protein synthesis, transcription, poly(A) RNA binding and ribosome (Figure S4).

The list of mRNAs identified in ribosomal footprinting in *Eif4e*^+/+^ and *Eif4e*^ki/ki^ mice is shown in Table S4. Many Wnt pathway components have long and structured 5′ UTRs (Untranslated Regions).^35^ Given the pronounced change in Wnt pathway mRNA translation, we further analyzed DTGs at 40 min post-HFS in the *Eif4e*^ki/ki^ vs.*Eif4e*^+/+^ mice, focusing on 5′ UTR mediated mechanisms. We find that the mRNAs of DTGs upregulated in *Eif4e*^+/+^ mice at 40 min post-HFS harbor 5′ UTRs which are significantly longer, and more complex (higher %GC and high folding free energy) and are enriched in uORF, TOP and PG4 motifs compared with downregulated DTG (Figure S7). Remarkably, HFS in *Eif4e*^ki/ki^ mice did not elicit the same response as *Eif4e*^+/+^ mice, displaying a loss of function phenotype with no significant differences neither in length, %GC, folding free energy nor in the incidence of uORF, TOP and PG4 motifs in upregulated DTG compared with downregulated (Figure S7). Taken together, these data suggest that eIF4E Ser209 phosphorylation is required in the DG for mRNA-specific translation during *in vivo* LTP and a major GO category regulated downstream is the Wnt signaling pathway.

### Synaptic activity-evoked eIF4E phosphorylation recruits atypical β-catenin to the translation initiation complex

In canonical Wnt signaling, β-catenin accumulates in the nucleus and activates transcription of Wnt target genes.^29^^,^^30^ However, a study in vascular smooth muscle cell cultures shows interaction of β-catenin with FMRP in the eIF4E cap-binding complex.^36^ In these cells, Wnt signaling triggers release of β-catenin from the complex, resulting in derepression of translation with nuclear accumulation of β-catenin.

**Figure 4:**
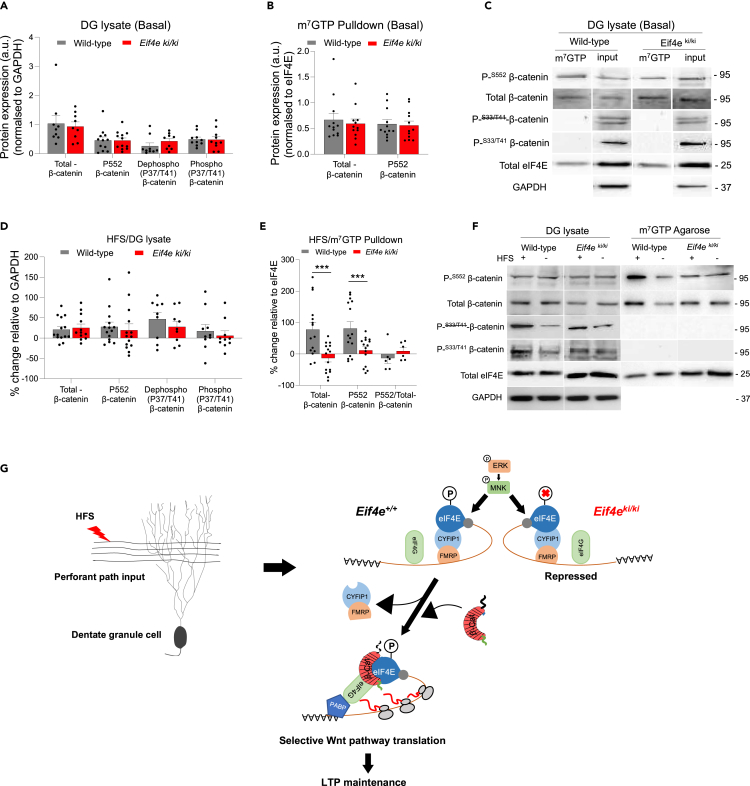
Synaptic activity-evoked eIF4E phosphorylation recruits (atypical) β-catenin to eIF4E cap complex in DG-LTP *in vivo* (A) Immunoblot analysis of total (T) and phosphorylated (P) β-catenin in DG lysates from naive mice (basal state). No significant difference between wild-type and *Eif4e*^ki/ki^ mice in expression of total β-catenin, P-552 (n = 9) β-catenin Arm domain (n = 12), P-37/41 β-catenin N-terminal region (n = 9), or non-37/41 phosphorylated N-terminal region (n = 10), normalized to GAPDH. Values are means +SEM (Multiple t-test). (B) Immunoblot analysis from m^7^GTP pulldown assays in DG lysates in naive mice (basal state). Densitometric values for Total and P-S552-β-catenin are normalized to values of m^7^GTP-bound eIF4E. Values in *Eif4e*^ki/ki^ mice were not significantly different from wild-type. (C) Representative immunoblots for panels (A) and (B). (D) Changes in the expression of total and phosphorylated β-catenin in DG lysate following HFS. Values are expressed in percent change relative to contralateral DG control. No significant difference was observed. (E) Immunoblot analysis of m^7^GTP pulldowns from DG lysates obtained 40 min post-HFS. Total- β-cat = 0.0013, P-^S552^ β-cat = 0.0074, P-^S552^/Total-β-cat =0.7040, (∗∗p < 0.001, ∗∗∗p < 0.0001, Multiple t-test). (Total- β-cat, n =12, P-^S552^ β-cat, n = 12, P-^S552^/Total-β-cat, n = 6). (F) Representative immunoblots for panels (D) and (E). HFS = high-frequency stimulation. (+) Ipsilateral DG, (−) Contralateral DG. Mean ± SEM and Student’s *t* test in Table S1. (G) Model of phospho-eIF4E-dependent translation of Wnt pathway in DG-LTP maintenance. (Left) HFS of medial perforant input to DG granule cells. (Right). In wild-type mice, synaptic activation by HFS stimulates ERK-MNK signaling and eIF4E phosphorylation on Ser209. Phosphorylation triggers discharge of eIF4E-binding proteins, both CYFIP1 and 4E-BP2, and recruitment of β-catenin (β-cat) to the eIF4E cap complex. Release of CYFIP1 together with its binding partner FMRP is depicted. The β-catenin that associates with eIF4E is atypical as its N-terminal region (indicated by dotted line) is not detected by specific antibodies. Phosphorylation of eIF4E is required for Wnt pathway translation as a class and underlies LTP maintenance. In *Eif4e*^ki/ki^ mice, ERK-MNK signaling is activated but loss of eIF4E phosphorylation prevents remodeling of the eIF4E complex, Wnt pathway translation, and LTP. See also Figure S7.

We aimed to determine whether β-catenin is part of the eIF4E-cap complex of adult DG, and, if so, whether it is regulated by LTP-inducing stimuli and Ser209 eIF4E phosphorylation. In DG from naive unstimulated mice, β-catenin was detected in m^7^GTP cap-pulldowns and lysate samples, with no significant difference in expression between *Eif4e*^ki/ki^ and wild-type mice (Figures 4A–4C). Thus, under basal conditions, β-catenin associates with eIF4E in a manner that does not depend on eIF4E phosphorylation. Following LTP induction, β-catenin in lysate samples from HFS-treated DG was increased 20% relative to the contralateral control, but there was no difference between genotypes (Figures 4D and 4F). In contrast in the cap-pulldown assays, HFS in wild-type mice elicited a significant mean 78.3% enhancement of β-catenin levels whereas no change was found in *Eif4e*^ki/ki^ mice (Figures 4E and 4F). These data demonstrate recruitment of β-catenin to eIF4E that is evoked by HFS of perforant path synapses, dependent on eIF4E phosphorylation, and functionally linked to LTP maintenance and enhanced translation of Wnt signaling pathway (model shown in Figure 4G).

β-catenin stability and transcriptional activity are regulated by phosphorylation. We therefore asked whether the β-catenin that associates with eIF4E represents a distinct form. Immunoblotting was done using antibodies recognizing critical phosphorylation sites on β-catenin’s N-terminal intrinsically disordered region and within its central Armadillo (Arm) repeats domain.^37^ In the absence of Wnt, GSK3-catalyzed phosphorylation of the β-catenin N-terminus (Ser33/Thr41) promotes ubiquitination and proteasomal degradation.^38^^,^^39^ In the presence of Wnt, dephosphorylation of the N-terminal residues prevents degradation, allowing accumulation of β-catenin in the nucleus to regulate transcription. We probed with antibodies specifically recognizing phosphorylated (Ser33/Thr41) or non-phosphorylated N-terminal epitopes. Robust signals were detected with both antibodies in DG lysates from naive animals, with no difference between genotypes (Figures 4A and 4C). Following LTP induction, enhanced expression of phosphorylated and non-phosphorylated N-terminal β-catenin were observed, but again there was no difference between knockin and wild-type (Figures 4D and 4F). Remarkably, immunoblots in cap-pulldown samples from naive and HFS-treated mice of both genotypes were negative for both phosphorylated and non-phosphorylated N-terminal β-catenin (Figures 4C and 4F). This could mean that β-catenin in the cap complex lacks the N-terminus or is modified in a way that prohibits binding of the specific antibodies. The fact that total β-catenin is reliably detected at the expected molecular mass (95 kDa) in all cap-pulldown and lysate samples shows that β-catenin is not proteolytically cleaved.

Phosphorylation of Ser552 in the Arm domain regulates protein-protein interactions, with enhanced phosphorylation decreasing partner binding and promoting nuclear accumulation.^30^^,^^37^^,^^40^^,^^41^ β-catenin Ser552 phosphorylation was detected in both cap-pulldown and lysate samples, but again, with no difference between genotypes at baseline (Figures 4A–4C). Following LTP induction, phospho-Arm was increased in HFS-treated DG relative to the contralateral DG in both genotypes (Figures 4D and 4F). Strikingly, in cap-pulldown samples, Ser552 phosphorylated β-catenin was significantly increased 78.3% above contralateral control in wild-type mice and this increase was abolished in *Eif4e*^ki/ki^ mice. However, normalization to total β-catenin showed that Ser552 phosphorylation state did not change (Figure 4E). Thus, HFS induces a phospho-eIF4E-dependent recruitment of β-catenin to eIF4E where it maintains constitutive levels of Ser552 Arm phosphorylation.

Finally, immunoblotting in DG lysates was done to assess changes in protein expression of select, translationally upregulated Wnt pathway targets. In wild-type mice, HFS significantly increased expression of the key Wnt receptor scaffolding protein, Dvl2, relative to contralateral control, whereas no significant change was observed in *Eif4e*^ki/ki^ mice (Figure S8). Expression of Frizzled class receptor 4 (Fzd4) or secreted frizzled-related protein 1 (Sfrp1) was unchanged, indicating differential impacts on protein expression in early LTP maintenance at 40 min post-HFS.

## Discussion

This study elucidates a mechanism and function for Ser209 eIF4E phosphorylation in translational control of DG-LTP *in vivo*. Biochemically, eIF4E phosphorylation is required for synaptic activity-evoked remodeling of the cap-binding complex. The remodeling is bidirectional, with discharge of the translational repressors CYFIP1 and 4E-BP2, and recruitment of β-catenin. In the first translational profiling analysis of DG-LTP, we find that phospho-eIF4E is specifically required for synaptic activity-induced translation of the Wnt pathway. Functionally, ablation of phospho-eIF4E does not alter basal synaptic transmission or LTP induction but inhibits the maintenance phase of LTP. Previous work demonstrated critical roles for Wnt signaling in LTP at excitatory synapses.^42^^,^^43^^,^^44^ Although β-catenin is known to mediate transcription downstream of Wnt, our results identify novel phospho-eIF4E-dependent recruitment of β-catenin to eIF4E and enhanced Wnt path translation specific to LTP maintenance.

Previous work demonstrated that acute pharmacological inhibition of MNK inhibits eIF4E phosphorylation and DG-LTP maintenance. However, MNKs have additional substrates which could impact translation and mRNA metabolism, including eIF4G, PSF (polypyrimidine tract-binding protein-associated splicing factor), and heterogeneous ribonucleoprotein A1.^19^^,^^20^^,^^31^^,^^45^ Here, we demonstrate that activation of ERK-MNK signaling and LTP induction are intact in *Eif4e*^ki/ki^ mice whereas stable maintenance of LTP is lost. Basal perforant path-evoked synaptic transmission and eIF4E protein-protein interactions were intact, with no difference between genotypes in eIF4E associated CYFIP1/FMRP, 4E-BP2, β-catenin, or eIF4G. Thus, the Ser209Ala Knockin mutation does not lead to compensatory upstream changes in ERK-MNK signaling or downstream remodeling of the eIF4E complex. Rather, our results demonstrate a crucial role for phospho-eIF4E in stimulus-evoked discharge of CYFIP1 and 4E-BP2 from eIF4E and enhanced loading of eIF4G to facilitate initiation. CYFIP1 can shuttle between the eIF4E complex and a Rac-WAVE1 complex involved in actin cytoskeletal remodeling and dendritic spine plasticity.^46^^,^^47^ If such shuttling occurs in LTP, disruption of CYFIP1 release may impact both translation and actin dynamics.

Wnts have broad and diverse functions in embryonic patterning and development, including neuronal dendrite development and synapse formation, and continue to function in activity-dependent synaptic plasticity in the adult brain.^30^^,^^42^^,^^48^ Neuronal activity-induced Wnt secretion and signaling through canonical β-catenin and the non-canonical planar cell polarity (PCP) and calcium pathways are implicated in LTP of excitatory synaptic transmission.^42^^,^^43^^,^^44^^,^^49^^,^^50^ Roles for Wnt signaling in trafficking of AMPA-type glutamate receptors and structural plasticity of dendritic spines have been identified.^42^^,^^51^ In DG-LTP, canonical Wnt/β-catenin signaling is associated with transcription of Wnt target genes.^7^^,^^43^ Our ribosome profiling analysis uncovered phospho-eIF4E-dependent translation of Wnt-4 and Wnt receptors and scaffolds (Dvl2, Lrp5, Fzd-2 and 4) as well as endogenous inhibitors of Wnt receptors (Sfrp1 and 2). Targeting of the Wnt pathway as a class suggests a coordinate regulation which could serve to amplify Wnt signaling in LTP.

Whether recruitment of β-catenin to eIF4E is directly involved in regulation of Wnt pathway translation remains to be determined. Our analysis of mRNA features indicates that phospho-eIF4E preferentially promotes translation of mRNAs with long, structured 5′ UTRs. However, as these features are not unique to Wnt family components, other factors must provide specificity. In the developing nervous system, β-catenin interaction with N-cadherin regulates dendritic spine plasticity.^52^^,^^53^N-cadherin stimulates Akt, which phosphorylates β-catenin Ser552.^54^ Phosphorylation of β-catenin Ser552 has been shown to regulate protein-protein interactions. We show that β-catenin in the eIF4E complex is Ser552 phosphorylated, but there is no change in phosphorylation state to support phosphorylation as a mechanism of recruitment. Under reducing conditions on SDS-PAGE gels, immunoblotting showed that antibodies specific for the N-terminal intrinsically disordered region reliably and clearly detect β-catenin in lysates, whereas cap-pulldown assays are blank. This suggests that β-catenin in the eIF4E complex is a unique form that has undergone a structural change or post-translational modification that prevents detection of the N-terminal epitope.

Translation control can be general, affecting translation in a global manner, or more specific, impacting only subsets of mRNAs.^55^^,^^56^ HFS in wild-type and *Eif4e*^ki/ki^ mice increased expression of numerous IEG mRNAs and enhanced translational efficiency in gene ontology categories synaptic regulation and plasticity. Of these, the Wnt pathway was the only category of DTGs completely dependent on phospho-eIF4E. We compared our list of phospho-eIF4E regulated DTGs in DG *in vivo* with FMRP-target genes^57^ and MNK1 targets identified using cortical neuronal cultures from MNK1 knockout mice^58^ (Figure S9). Although the preparations and methods are different, the main conclusion seems to be that there are few common mRNAs (less than 5%). This reinforces the importance of phospho-eIF4E in specific translation during LTP in the DG. DG-LTP consolidation has mechanistically distinct early and late phases of translation with different targets. We concentrated on the critical, early stage of translation (40 min post-HFS) and recognize that new patterns may emerge at later time points.

In cancer models, phospho-eIF4E regulates translation of targets involved in oncogenic transformation.^59^^,^^60^ In the suprachiasmatic nucleus, phospho-eIF4E regulates translation of clock genes (*Per1* and *Per2*) involved in circadian rhythms^61^ and in dorsal root ganglion phospho-eIF4E promotes translation of *bdnf* involved in hyperalgesia and nociceptive transmission.^62^ A recent ribosome footprinting analysis of forebrain tissue of adult *Eif4e*^ki^ mice revealed regulation of mRNAs involved in inflammation (IL-2 and TNFα) and organization of extracellular matrix (Prg2, Mmp9, Adamts16, Acan).^26^^,^^63^ Behavioral analyses of *Eif4e*^ki/ki^ mice have revealed a role for phospho-eIF4E in regulation of depression-like behavior.^26^ Collectively, evidence suggests that phospho-eIF4E regulates translation in a region- and stimulus-specific manner.

A previous analysis of the Schaffer collateral-CA1 pathway in hippocampal slices from *Eif4e*^ki/ki^ mice showed normal basal transmission and LTP induction as well as normal late phase LTP maintenance (recorded for 2 h).^26^ The ability to generate stable LTP in CA1 but not DG in *Eif4e*^ki/ki^ mice could reflect differences in the balance and timing of mTORC1 and MNK-dependent translation in these circuits. LTP maintenance in CA1 requires mTORC1 signaling, which triggers removal of 4E-BP2.^64^^,^^65^^,^^66^^,^^67^ Stable CA1-LTP induced by HFS or BDNF application is blocked by the mTORC1 inhibitor rapamycin.^66^^,^^68^ In DG-LTP, inhibition of mTORC1 signaling by rapamycin does not affect LTP induction or maintenance.^27^^,^^28^ It is therefore possible that ablation of phospho-eIF4E is compensated by mTORC1 signaling in region CA1, whereas MNK regulation predominates in DG-LTP and is not developmentally compensated in *Eif4e*^ki/ki^ mice.

A recent study showed that inhibition of eukaryotic elongation factor 2 (eEF2) phosphorylation by conditional deletion of eEF2 kinase dramatically increases neurogenesis in the adult DG, enhances DG-dependent cognitive functions and decreases depression-like behavior.^69^ The regulation of neurogenesis is specifically linked to proteostasis of mature DG granule cells, with increased expression of neurogenesis-related proteins (decorin, vimentin). The phenotype in *Eif4e*^ki/ki^ mice is very different, with normal DG neurogenesis and hippocampal-dependent memory function, whereas depression-like behavior is increased.^26^^,^^70^ Conceivably, eEF2 has a primary function in supporting neurogenesis-related translation whereas phosho-eIF4E supports synaptic plasticity of pre-existing inputs to mature granule cells. These different forms of translational control may cooperate in DG functions such as regulation of depression-like behavior.

### Limitations of the study

Although genotypes did not differ in basal assembly of the translation initiation complex and specific defects in stimulus-evoked translation and plasticity were found in *Eif4e*^ki/ki^ mice, compensatory developmental changes in the Knockin mice cannot be ruled out. The study uncovers functions for eIF4E phosphorylation *in vivo* but does not address the molecular mechanisms underlying discharge of repressors and recruitment of β-catenin. *In vitro* studies are needed to elucidate the molecular function β-catenin in complex with eIF4E.

## STAR★Methods

### Key resources table

**Table:** 

REAGENT or RESOURCE	SOURCE	IDENTIFIER
**Antibodies**		

Arc; Mouse; 1:500	Santacruz biotechnology	Cat# sc-17839; RRID: AB_641123
CYFIP1; Rabbit; 1:1000	Upstate technology	Cat#07-531; RRID: AB_390148
FMRP; Rabbit; 1:1000	Abcam	Cat#17722; RRID: AB_10001160
4E-BP2; Rabbit; 1:1000	Cell signalling	Cat# 2845, RRID: AB_10699019
GAPDH; Mouse; 1:500	Santacruz biotechnology	Cat# sc-32233, RRID: AB_627679
P-eIF4E; Rabbit; 1:1000	Cell signalling	Cat# 9741, RRID: AB_331677)
eIF4E; Rabbit; 1:1000	Cell signalling	Cat# 9742, RRID: AB_823488
eIF4G; Rabbit; 1:1000	Cell signalling	Cat# 2498, RRID: AB_2096025
p-MNK1 Thr197/202; Rabbit; 1:500	Cell signalling	Cat# 2111, RRID: AB_2266303
MNK1; Rabbit; 1:1000	Cell signalling	Cat# 2195, RRID: AB_2235175)
4E-BP1 Thr37/46; Rabbit; 1:500	Cell signalling	Cat# 2855, RRID: AB_560835
RPL13a ; Rabbit; 1:1000	Cell signalling	Cat# 2765, RRID: AB_916223
EPRS; Rabbit; 1:1000	Abcam	Cat# ab31531, RRID: AB_880047
Total-β-catenin; Rabbit; 1:1000	Millipore	Cat# AB19022, RRID: AB_2088263
P-β-catenin S552; Rabbit; 1:1000	Cell signalling	Cat# 5651, RRID: AB_10831053
P-β-catenin S33/T41; Rabbit; 1:1000	Cell signalling	Cat# 9561, RRID: AB_331729)
Active-β-catenin S33/T41; Rabbit; 1:1000	Millipore	Cat# 05-665, RRID: AB_309887
Anti-Fzd4; Rabbit; 1:1000	Nordic Biosite	Cat# ASJ-YDM4RO
Sfrp1; Rabbit; 1:1000	Fisher scientific	Cat# 16304905
Dvl2; Rabbit; 1:1000	Fisher scientific	Cat# PA5-17471, RRID:AB_10982147

**Deposited data**		

RNA-seq data Processed next-generation sequencing data, in accordance with the MINSEQE standards, as enforced by recommended, datatype-specific repositories (e.g., GEO)	FORTH-Gkogkas – deposited to Mendeley	Gkogkas, Christos (2023), “Patil et al. iScience 2023 - Ribosome Profiling”, Mendeley Data, V1, https://doi.org/10.17632/6dyxx4prmg.1
Western blots	University of Bergen, Department of Biomedicine, Bramham lab – deposited to Mendeley data.	Patil, Sudarshan (2023), “Original blots”, Mendeley Data, V1, https://doi.org/10.17632/nhb7zd3gcw.1

**Experimental models: Organisms/strains**		

eIF4E S209A knockin mice	McGill University Canada (Sonenberg Lab)	Furic et al.

**Software and algorithms**		

Ribosome profiling analysis pipeline	FORTH Greece, Gkogkas lab	Simbriger et al.^71^
DAVID web server	https://david.ncifcrf.gov/	Sherman et al.^72^
IPA	QIAGEN	https://digitalinsights.qiagen.com/products-overview/discovery-insights-portfolio/analysis-and-visualization/qiagen-ipa/

### Resource availability

#### Lead contact

Further information and requests for resources and reagents should be directed to and will be fulfilled by the lead contact, Clive Bramham (clive.bramham@uib.no).

#### Materials availability

This study did not generate new unique reagents.

### Experimental model and subject details

#### Animals

*Eif4e*^*S209A*^ mice previously described^63^ were used. *In vivo* electrophysiological experiments were carried out on 90 male homozygous *Eif4e*^S209A/S209A^ mice (*Eif4e*^ki/ki^), heterozygous *Eif4e*^ki/+^mice, and wild-type C57BL/6 mice (*Eif4e*^+/+^) (Taconic Europe, Ejby, Denmark), weighing 25-30 g. Mice were bred and housed in their home cages. Room temperature (22°C±1°C) and relative humidity (46±5%) was maintained. Mice had free access to water and autoclaved standard rodent diet (SDS, England; RMI-E) and were maintained on a 12 h light/dark cycle. This research is approved by Norwegian National Research Ethics Committee in compliance with EU Directive 2010/63/EU, ARRIVE guidelines. Persons involved in the animal experiments have approved Federation of Laboratory and Animal Science Associations (FELASA) C course certificates and training.

### Method details

#### Antibodies used

Antibodies used for immunoblotting are listed in Table S1.

#### *In vivo* electrophysiology in mice

Electrophysiology methods are as described^28^ with minor modifications. Adult mice (12-weeks old) were anesthetized with urethane (injected i.p. 1.2 g/kg), which was supplemented throughout surgery and recording as required. Mice were placed in a stereotaxic frame and body temperature was maintained at 37°C. In one hemisphere only, a bipolar stimulation electrode (NE-200, 0.5 mm tip separation, Rhodes Medical Instruments, Wood hills, CA) was positioned for unilateral stimulation of the perforant path (3.8 mm posterior to bregma, 2.5 mm lateral to midline, and 1.6 mm depth from the brain surface) while an insulated tungsten recording electrode (0.075 mm; A-M Systems) was positioned in the DG hilar region (2 mm posterior to bregma, 1.5 mm lateral to the midline, and 1.5 – 1.7 mm depth from the brain surface). The recording electrode was lowered into the brain in 0.1 mm increments while monitoring the laminar profile of the response waveform evoked by a 400 μA test-pulse stimulus. To generate input/output (I/O) curves, 7 stimulus intensities ranging from 80 μA to 400 μA were applied in randomized sequence. After generating an I/O curve, a stable 20 min baseline of evoked potentials was recorded using test-pulses of 0.1 ms pulse-width applied at 0.033 Hz. The test-pulses intensity produced a population spike of 30% of maximum. The high-frequency stimulation (HFS) protocol consisted of four trains of stimuli applied with an interval of 10 sec; each train had 15 pulses at 200 Hz (pulse-width 0.1 ms). The stimulus intensity for HFS was twice that used for baseline recordings. After HFS, test-pulse evoked responses were recorded for 40- and 180-min. After recordings were completed, the electrodes were removed, the animal was sacrificed, and the dentate gyri were micro-dissected and immediately frozen on dry-ice for later use. The maximal slope of the initial rising phase of the fEPSP and population spike amplitude were measured, and changes post-HFS were expressed in percent of baseline. In the electrophysiological experiments for ribosome profiling, mice received the standard LTP protocol consisting of low-frequency test-pulse stimulation (LFS) and HFS. DG tissue was collected at 40 min post-HFS. To control for effects of test-pulse stimulation, a control group received LFS only for 40 min.

#### Tissue dissection and sample preparation

At the end of electrophysiological recording, ipsilateral and contralateral dentate gyri were rapidly dissected on ice and homogenized in buffer containing 50 mM Tris, 100 mM NaCl, 1 mM EDTA, NP-40 0.5%, 1 mM dithiothreitol, 1 mM Na_3_VO_4_, 50 mM NaF, and 1× protease inhibitor cocktail from Roche #11836170001. Homogenization was performed manually with 10–12 gentle strokes in a tissue grinder and the homogenate was centrifuged for 10 min at 14000 x g at 4°C. The BCA protein assay was used for quantitation of total protein in lysates using BSA as a protein standard. (23225, Pierce, Thermofisher Scientific, Waltham, USA). Homogenates were stored at -80°C until use.

#### m^7^GTP pulldown assays

m^7^GTP pulldown assays have been described in detail elsewhere.^26^^,^^28^ In brief, 250-300 μg of protein lysate together with 30 μl of 7-methyl GTP-agarose beads (Jena bioscience #AC-141) were incubated for 90 min at 4°C. Beads were washed three times with m^7^GTP lysis buffer and bound proteins were separated by SDS-PAGE (10% gels or 4-15% gradient gels). Immunoblotting was carried out as described below.

#### SDS–PAGE and immunoblotting

Samples from lysates (15 μg) and m^7^GTP pull-down assays were heated at 95°C for 5 min in Laemmli sample buffer (Bio-Rad Laboratories, Hercules, USA) and resolved in 10% or 4-15% gradient SDS-PAGE gels. Proteins were transferred to nitrocellulose membranes (Biorad, # 162-0112) which were then blocked with 5% BSA, probed with antibodies and developed using chemiluminescence reagents (Biorad, #1705061). The blots were scanned using Gel DOC XRS+ (BIO RAD) and densitometric analyses were performed with Image J software (NIH, Bethesda, MD). Blots treated with phospho-specific antibody were stripped with 100 mM 2-mercaptoethanol, 2% SDS and 62.5 mM Tris-HCl, pH 6.8 at 50°C for 45 min, washed, blocked and re-probed with antibody recognizing total protein. Densitometric values for total proteins were normalized to GAPDH in lysate samples and to eIF4E in m^7^GTP pull-downs. Values from the HFS-treated DG were expressed in percent change relative to contralateral control DG run on the same gel. Quantification for all bands was performed in the linear range of detection.

#### Ribosome profiling (RP) & bioinformatics analysis

Tissue was processed using the TruSeq Ribo Profile (Mammalian) Kit (Illumina), according to the manufacturer’s instructions. Briefly, tissue was homogenised in Mammalian Polysome Buffer (Illumina) supplemented with DNAse I (10 U/mL), 1% Triton X-100, 1% NP-40, 1 mM DTT and cycloheximide (100 μg/mL). Half of the lysate was used for mRNA extraction (total mRNA) while the remaining fraction was digested with TruSeq Ribo Profile Nuclease so that only the mRNA fragments protected by ribosomes were recovered (footprints). Both samples (footprints and total mRNA) went through a ribosomal RNA removal step using the Ribo-Zero Gold (Human/Mouse/Rat) Kit (Illumina). The footprint samples were then purified on a 15% TBE-Urea polyacrylamide gel (ThermoFisher Scientific) to select for fragments of 28-30 nucleotides. The total mRNA samples were heat-fragmented, according to the TruSeq Ribo Profile protocol, to yield small RNA fragments. Footprints and total mRNA fragments were used to prepare small RNA libraries, using the TruSeq Ribo Profile Kit, and were sequenced on an Illumina HiSeq 2500 System, at the Edinburgh Genomics facilities. Bioinformatics analysis was performed as previously described (Amorim et al., 2018).^26^ Translational Efficiency (TE) was calculated as the ratio between RPKM of footprints and RPKM of total mRNA for each gene. Data was filtered to include only Differentially Translated Genes (DTGs) that meet the following criteria: FDR<0.05, p-value <0.05, and -1<Log_2_(TE)>0.585.

#### Gene ontology and pathway analysis

Gene Ontology (GO) and Pathway Analysis were performed using the online tool DAVID (Database for Annotation, Visualization and Integrated Discovery^73^ version 6.8) and the Ingenuity Pathway Analysis Software (IPA; Qiagen; version 42012434), respectively. Differentially translated genes were submitted to IPA and subjected to Core Analysis with analysis parameters set to include Direct and Indirect Interactions and Experimentally Observed data only. For further analysis of relevant Canonical Pathways, a Molecular Activity Predictor (MAP) analysis was applied based on the differentially regulated genes belonging to each individual pathway. For GO analysis, filtered gene lists split to highlight genes differentially upregulated or downregulated in each dataset were individually submitted to DAVID and GO annotation gathered for Biological Function, Molecular Function and Cellular Component.

### Quantification and statistical analysis

Group values are reported as mean ± SEM. Statistical comparisons were calculated with the Two-way repeated measures ANOVA with Bonferroni multiple-comparison test or multiple T-test with Holm-Sidak method using GraphPad Prism 8.02. Significance level was set *a priori* at p < 0.05. Statistical analysis data is summarized in Table S1, and also provided in the figure legends.

## Data Availability

RNA-seq data will be deposited at GEO and made publicly available as of the date of publication. Accession numbers are listed in the key resources table. Original western blot images will be deposited at Mendeley and will be publicly available. This paper does not report original code. Any additional information required to reanalyze the data reported in this paper is available from the lead contact upon request.

## Supplemental information

Document S1. Figures S1–S9 and Table S1

Table S2. Differential expression and translation of genes in HFS-treated wild-type mice: comparison with contralateral and LFS-treated control groups, related to Figure 3

Table S3. Gene Ontology (GO) and pathway analysis, related to Figure 3

Table S4. Differential expression and translation of genes in HFS-treated wild-type *Eif4e*^+/+^ mice and *Eif4e*^ki/ki^ mice, related to Figure 3

Table S5. List of differentially translated mRNAs in GO categories canonical Wnt signaling, actin cytoskeletal regulation, and ribosomal proteins, related to Figure 3
