# Supplementary material for: eIF4E phosphorylation recruits β-catenin to mRNA cap and promotes Wnt pathway translation in dentate gyrus LTP maintenance
Source: iScience. 2023 Apr 15;26(5):106649. doi: 10.1016/j.isci.2023.106649 (PMC10214474; doi:10.1016/j.isci.2023.106649)
Supplement: Document S1. Figures S1–S9 and Table S1 [file mmc1.pdf]

iScience, Volume *26*

Supplemental information

eIF4E phosphorylation recruits β-catenin to mRNA cap and promotes Wnt pathway translation in dentate gyrus LTP maintenance

Sudarshan Patil, Kleanthi Chalkiadaki, Tadiwos F. Mergiya, Konstanze Krimbacher, Inês S. Amorim, Shreeram Akerkar, Christos G. Gkogkas, and Clive R. Bramham

Supplemental Figure S1

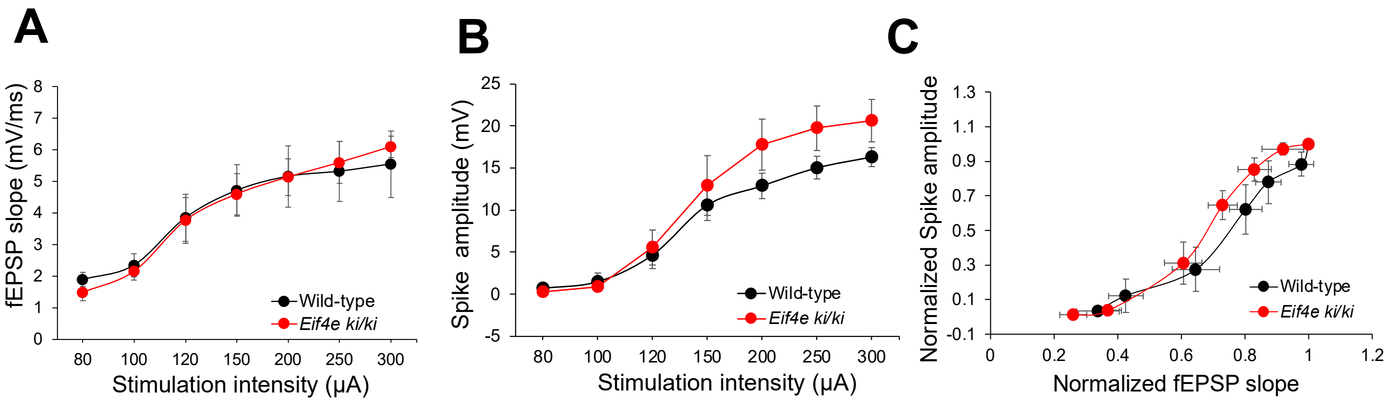

Supplemental Figure S2

A

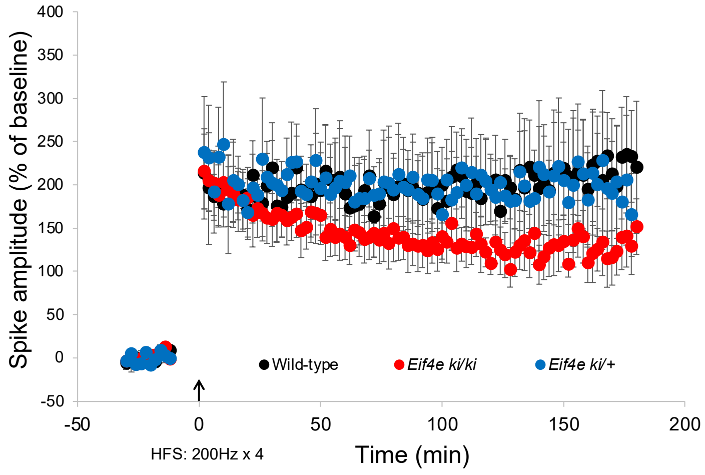

B

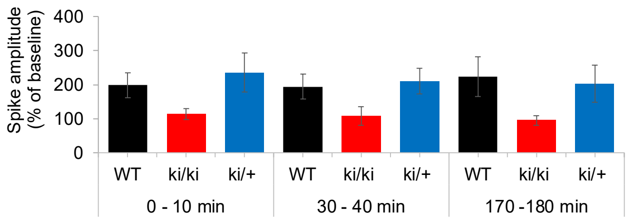

Supplemental Figure S3

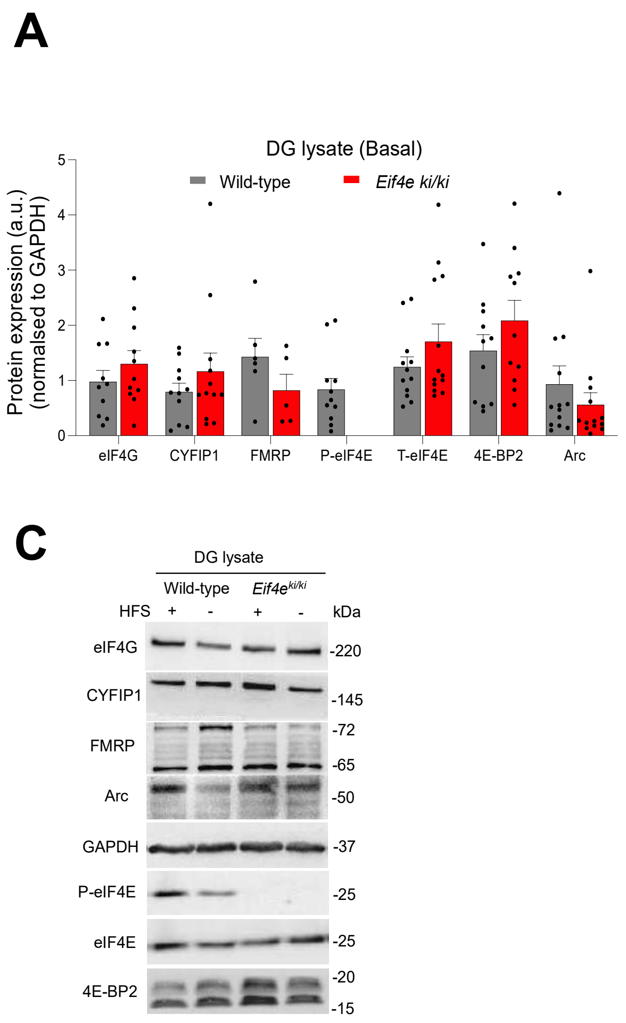

Supplemental Figure S4

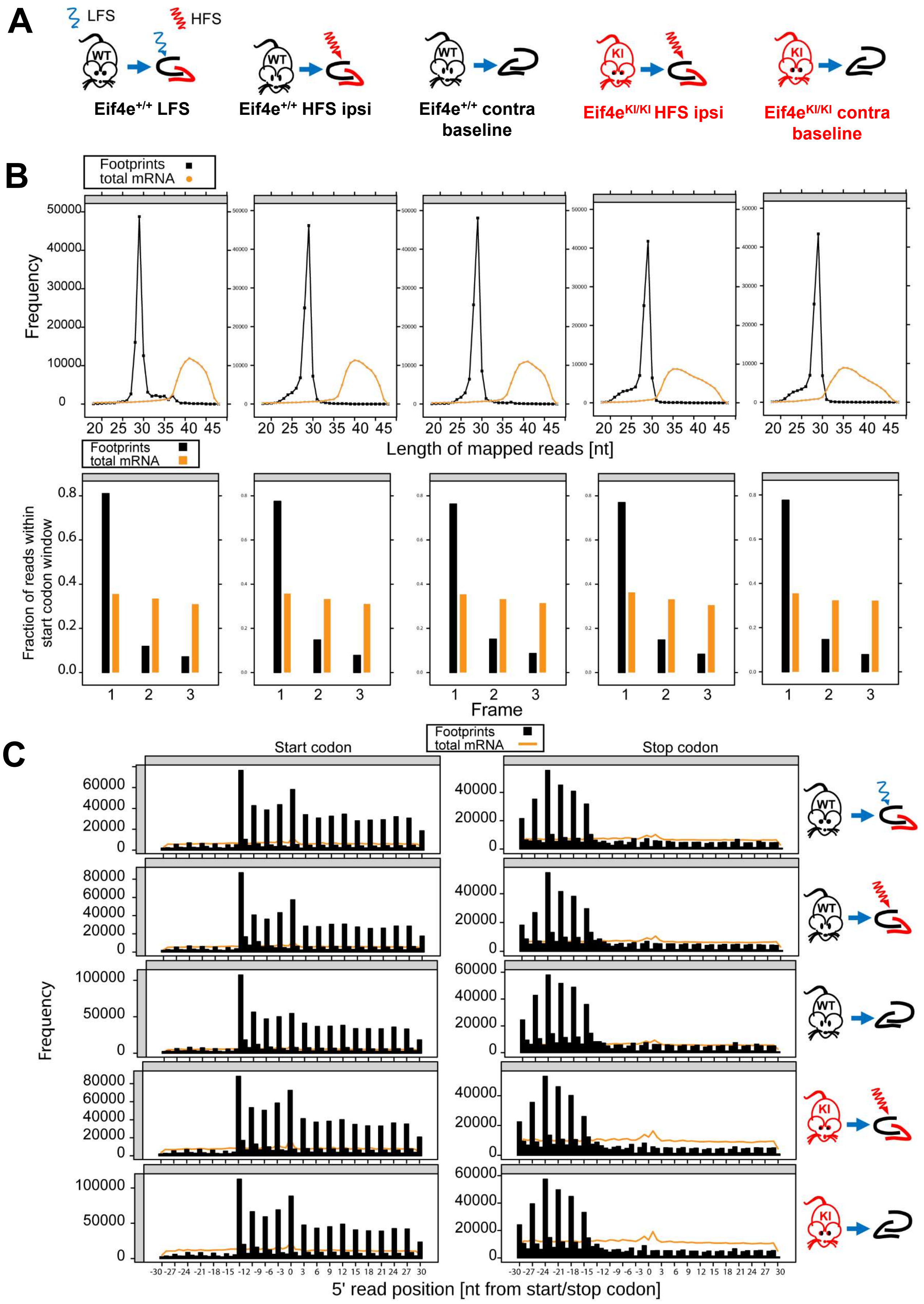

Supplemental Figure S5

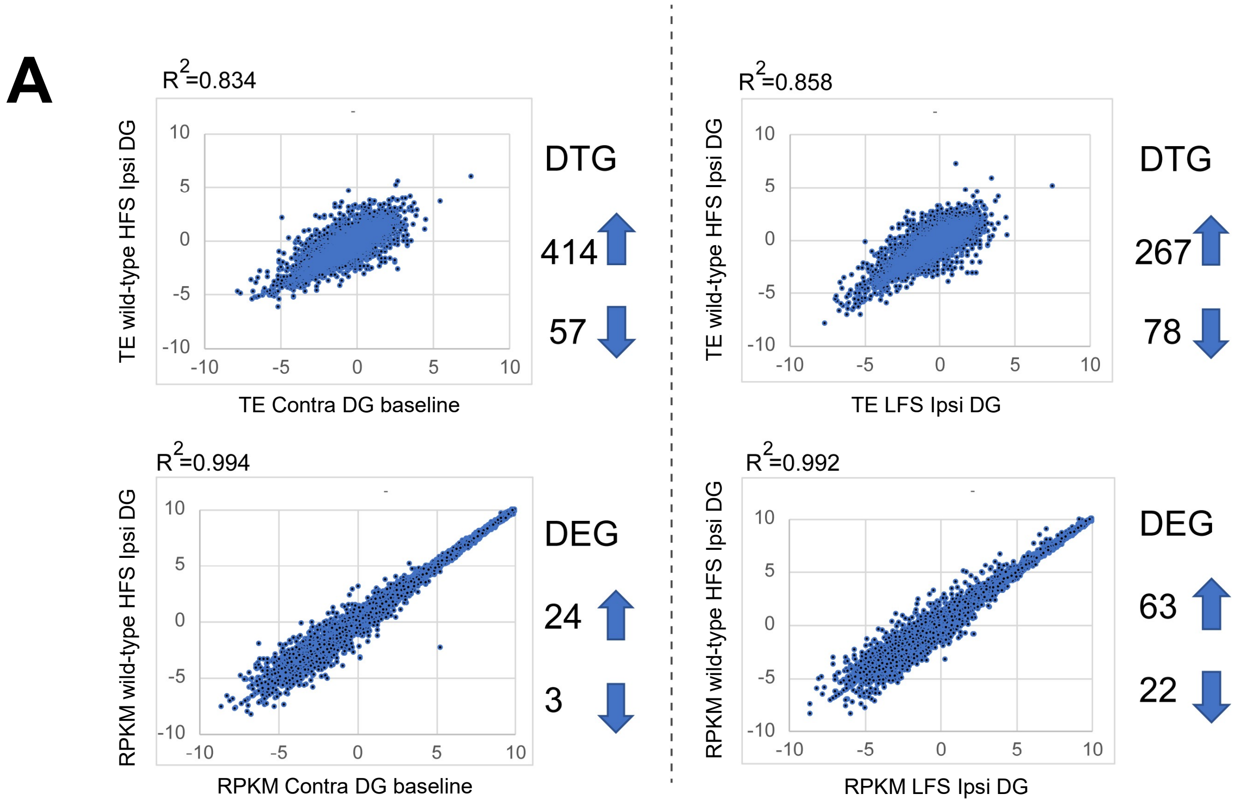

B

	DTG IPA Canonical pathways	DEG IPA Canonical pathways
**Wild-type HFS Ipsi DG vs Contra DG**	14-3-3-mediated Signaling	GP6 Signaling Pathway
	AMPK Signaling	Hepatic Fibrosis/Hepatic Stell. Cell Act.
	Actin Cytoskeleton Signaling	Growth Hormone Signaling
**Wild-type HFS Ipsi DG vs LFS Ipsi DG**	AMPK Signaling	Glucocorticoid Receptor Signaling
	Actin Nucleation by ARP-WASP Complex	Neuroinflammation Signaling Pathway
	Agranulocyte Adhesion and Diapedesis	Sirtuin Signaling Pathway

Supplemental Figure S6

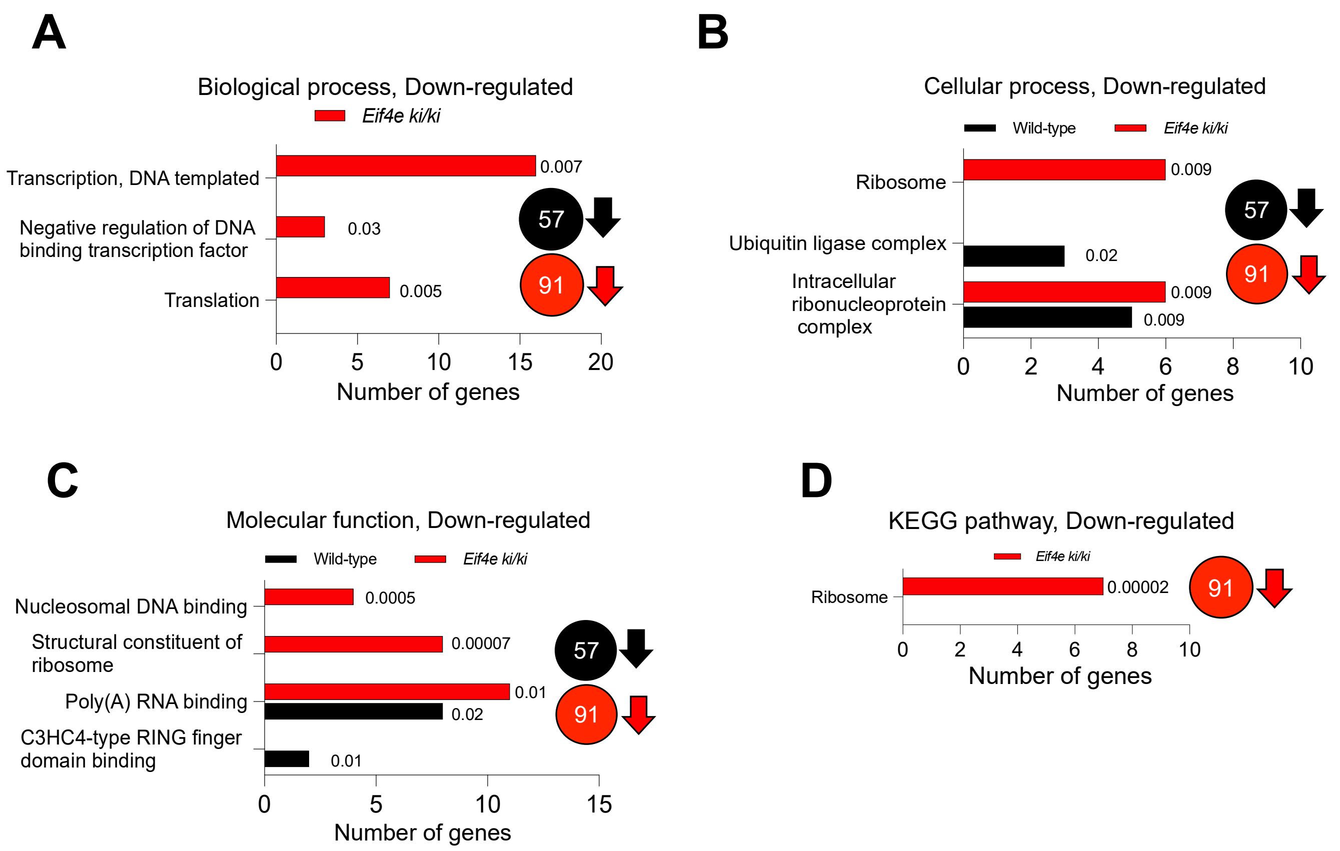

Supplemental Figure S7

A

	5' UTR analysis DTGs DOWN vs UP	
Feature	WT 40 min HFS	KI 40 min HFS
length	*	X
GC content	***	*
Gibbs Free energy	***	X

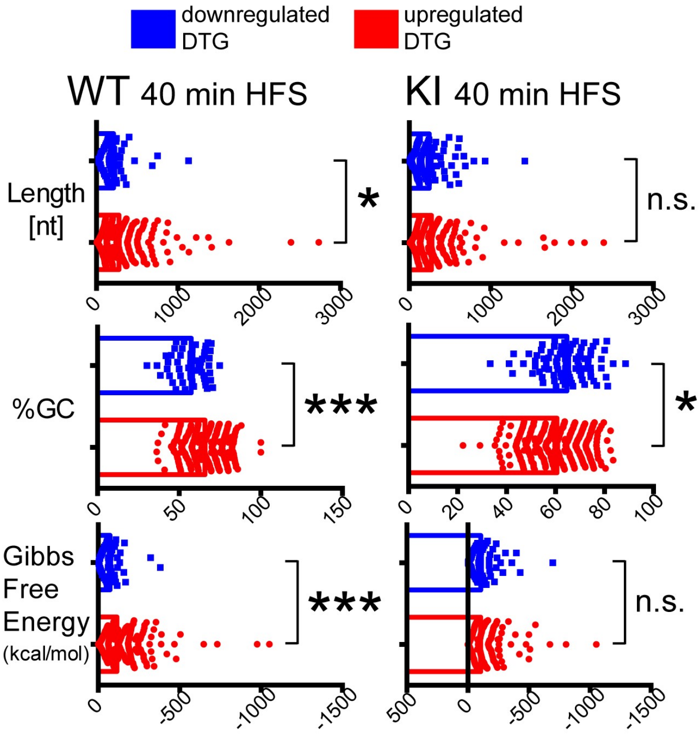

B

	5' UTR motifs DTGs DOWN vs UP	
Feature	WT 40 min HFS	KI 40 min HFS
uORF	*	X
TOP	*	X
IRES	X	*
PG4	*	X

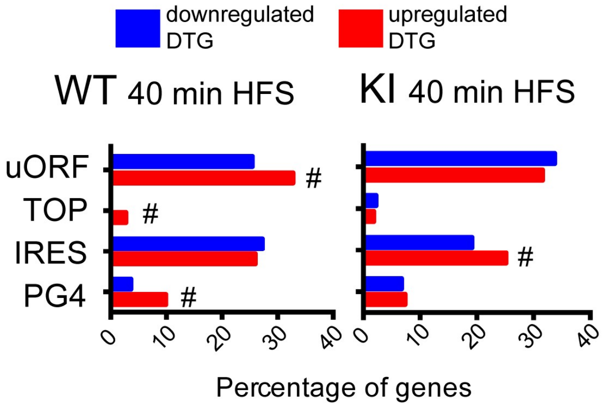

Supplemental Figure S8

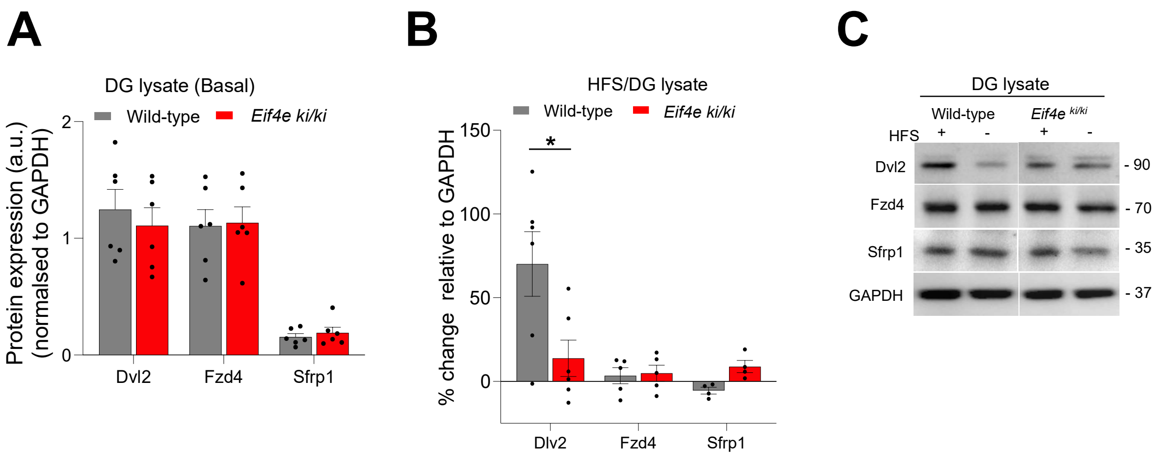

Supplemental Figure S9

A

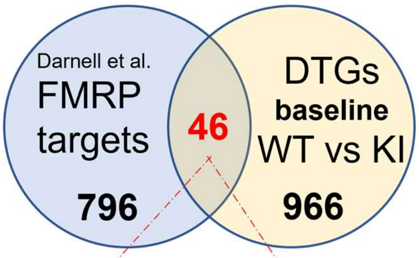

Shank1	Celsr3
Kif5a	Zc3h7b
Nlgn2	Lmtk3
Lingo1	Zfhx2
Grik5	Hdac5
Zmiz2	Tshz1
Dlgap3	Dtx1
Ski	Lrrn2
Gm996	Shank2
Lars2	Kcnc3
Cdk5r2	Bcl9l
Prr12	Pkd1
Cbx6	Mfhas1
Atn1	Tmem132a
Bcr	Nlgn3
Chst2	Crtc1
Igsf9b	Hdac4
Ttbk2	C2cd2l
Psd	Dot1l
Syt7	B3gat1
Hivep3	Synpo
Bzrap1	Jph4
Ttyh3	Camk2n1

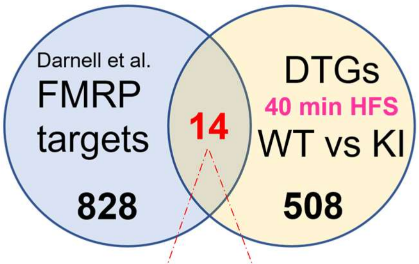

Pip5k1c
Git1
Kcnb1
Ckb
Zfp704
Calm3
Hcn2
Ppp1r9b
Kcnc3
Lrp3
Fscn1
Tubb3
Agpat3
Grm4

B

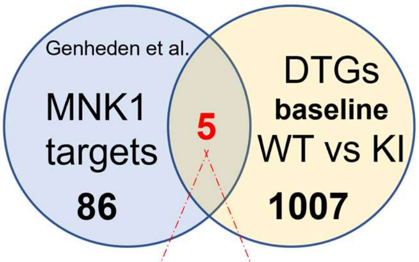

Sfrp1
Gps1
Atat1
Kif5a
Fosb

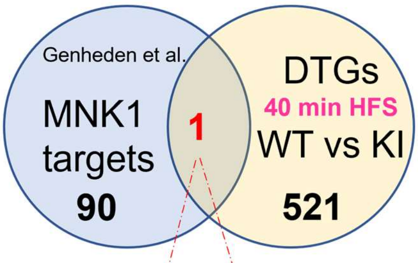

Flna

Supplementary Table S1. Statistical analysis.

Figure	Panel	mean± SEM	Group size	Statistics	Comparison	P-value
**1**	A					
	B	**Wild-type Vs. *Eif4e*$^{Ki/Ki}$**<br>30 min= 26.9 Vs 10.6<br>120 min= 29.1 Vs 5.6<br>180 min= 28.9 Vs 3.4	Wild-type = 10<br>*Eif4e*$^{Ki/Ki}$ = 8	Student t-test.		**Wild-type Vs. *Eif4e*$^{Ki/Ki}$**<br>30 min= 0.00037<br>120 min= 0.00016<br>180 min= 2.08^{-05}
		**Wild-type** = 26.9 10.6<br>*Eif4e*$^{Ki/Ki}$ = 16.3 3.0	Wild-type =10<br>*Eif4e*$^{KI/+}$ = 7	Student t-test.		**Wild-type Vs. *Eif4e*$^{KI/+}$**<br>30 min= 0.022<br>120 min= 0.6393<br>180 min= 0.9089
**2**	B	**Wild-type Vs. *Eif4e*$^{Ki/Ki}$**<br>P-MNK1/2 = 0.5Vs 0.7<br>T-MNK1/2 = 1.8 Vs 1.5<br>P-ERK1/2 = 2.9 Vs 2.3<br>T-ERK1/2 = 1.7 Vs 1.5	P-MNK1/2 = 7<br>T-MNK1/2 = 11<br>P-ERK1/2 = 6<br>T-ERK1/2 = 6	Multiple t-test.	Holm-Sidak Method	**Wild-typeVs. *Eif4e*$^{Ki/Ki}$**<br>P-MNK1/2= 0.4159<br>T-MNK1/2= 0.7122<br>P-ERK1/2 = 0.3978<br>T-ERK1/2 = 0.5933
	C	**Wild-type Vs. *Eif4e*$^{Ki/Ki}$**<br>eIF4G = 0.61 Vs 0.59<br>CYFIP1= 0.50 Vs 0.53<br>FMRP= 0.82 Vs 0.74<br>T-BP2= 1.05 Vs 1.39	eIF4G = 13<br>CYFIP1= 11<br>FMRP = 8<br>T-BP2 = 11	Multiple t-test.	Holm-Sidak Method	**Wild-type Vs. *Eif4e*$^{Ki/Ki}$**<br>eIF4G = 0.8907<br>CYFIP1= 0.8650<br>FMRP= 0.7343<br>T-BP2= 0.1518
	D	**Wild-type Vs. *Eif4e*$^{Ki/Ki}$**<br>P-/T-ERK1/2 = 86.8<br>P-/T-ERK1/2 = 64.93	Wild-type = 6<br>*Eif4e*$^{Ki/Ki}$ = 6	Student t-test.	Holm-Sidak Method	**Wild-type Vs. *Eif4e*$^{Ki/Ki}$**<br>P-/T-ERK1/2= 0.7507
	E	**Wild-typeVs. *Eif4e*$^{Ki/Ki}$**<br><br>P-/T-MNK1 = 26.2<br>P-/T-MNK1 = 24.1	P-MNK1 = 12<br>T-MNK1 = 10	Student t-test.	Holm-Sidak Method	**Wild-type Vs. *Eif4e*$^{Ki/Ki}$**<br>P-/T-MNK1 = 0.5026
	F	**Wild-typeVs. *Eif4e*$^{Ki/Ki}$**<br>eIF4G = 54.5 Vs -21.5<br>CYFIP1= -43.1 Vs 29.4<br>FMRP= -27.6 Vs 23.0<br>T-BP2= -14.7 Vs 31.4	eIF4G = 13<br>CYFIP1= 12<br>FMRP= 7<br>T-BP2= 11	Multiple t-test.	Holm-Sidak Method	**Wild-type Vs. *Eif4e*$^{Ki/Ki}$**<br>eIF4G = 0.00017<br>CYFIP1= 0.00004<br>FMRP= 0.008<br>T-BP2= 0.0005
**4**	A	**Wild-type Vs. *Eif4e*$^{Ki/Ki}$**<br>P-^{S552} β-cat = 0.46 Vs 0.45<br>Total- β-cat = 1.0 Vs 0.93<br>P-~~$^{S33/T41}$~~β-cat = 0.26 Vs 0.43<br>P-$^{S33/T41}$β-cat = 0.49 Vs 0.48	P-^{S552}β-cat = 12<br>Total-β-cat = 9<br>P-~~$^{S33/T41}$~~β-cat =10<br>P-$^{S33/T41}$β-cat = 9	Multiple t-test.	Holm-Sidak Method	**Wild-type Vs. *Eif4e*$^{Ki/Ki}$**<br>P-^{S552} β-cat = 0.9503<br>Total- β-cat = 0.7482<br>P-~~$^{S33/T41}$~~ β-cat = 0.2510<br>P-$^{S33/T41}$ β-cat = 9.9314

	B	**Wild-type Vs. *Eif4e*$^{Ki/Ki}$**<br>P-^{S552} β-cat = 0.37 Vs 0.40<br>Total- β-cat = 0.56 Vs 0.47	P-^{S552}β-cat = 12<br>Total-β-cat = 12	Multiple t-test.	Holm-Sidak Method	**Wild-type Vs. *Eif4e*$^{Ki/Ki}$**<br>P-^{S552} β-cat = 0.8053<br>Total- β-cat = 0.6198
	D	**Wild-type Vs. *Eif4e*$^{Ki/Ki}$**<br>P-^{S552} β-cat = 26.0 Vs 20.9<br>Total- β-cat = 23.4 Vs 6.5<br>P-$^{S33/T41}$β-cat = 47.0 Vs 27.8<br>P-$^{S33/T41}$β-cat = 17.4 Vs 6.1	P-^{S552} β-cat = 12<br>Total- β-cat = 9<br>P-$^{S33/T41}$β-cat =10<br>P-$^{S33/T41}$β-cat = 9	Multiple t-test.	Holm-Sidak Method	**Wild-type Vs. *Eif4e*$^{Ki/Ki}$**<br>P-^{S552} β-cat = 0.8230<br>Total- β-cat = 0.4362<br>P-$^{S33/T41}$ β-cat = 0.3847<br>P-$^{S33/T41}$ β-cat = 0.5730
	E	**Wild-type Vs. *Eif4e*$^{Ki/Ki}$**<br>Total- β-cat = 78.3 Vs -13.3<br>P-^{S552} β-cat = 81.2 Vs 11.8<br><br>P-^{S552} /Total- β-cat=101.8Vs 114.4	Total- β-cat = 12<br>P-^{S552} β-cat = 12<br><br>N=6	Multiple t-test.	Holm-Sidak Method	**Wild-type Vs. *Eif4e*$^{Ki/Ki}$**<br>Total- β-cat = 0.0013<br>P-^{S552} β-cat = 0.0074<br>P-^{S552} /Total-β-cat =0.7040
**S1**	A	**Wild-type Vs. *Eif4e*$^{Ki/Ki}$**<br>1. 1.889 Vs 1.484<br>2. 2.344 Vs 2.152<br>3. 3.842 Vs 3.759<br>4. 4.707 Vs 4.589<br>5. 5.332 Vs 5.127<br>6. 5.634 Vs 5.588<br>7. 5.863 Vs 6.088	Wild-type= 4<br>*Eif4e*$^{KI/Ki}$ = 4	Two-way ANOVA		**Wild-type Vs. *Eif4e*$^{Ki/Ki}$**<br>0.9994
	B	**Wild-type Vs. *Eif4e*$^{Ki/Ki}$**<br>1. 0.7027 Vs 0.2753<br>2. 1.482 Vs 0.8794<br>3. 4.642 Vs 5.533<br>4. 10.59 Vs 12.95<br>5. 12.89 Vs 17.78<br>6. 15.03 Vs 19.74<br>7. 16.33 Vs 20.61	Wild-type= 4<br>*Eif4e*$^{KI/Ki}$ = 4	Two-way ANOVA		**Wild-type Vs. *Eif4e*$^{Ki/Ki}$**<br>0.5977
	C	**Wild-type Vs. *Eif4e*$^{Ki/Ki}$**	Wild-type= 4<br>*Eif4e*^{KIKi} = 4	Three-way ANOVA		**Wild-type Vs. *Eif4e*$^{Ki/Ki}$**<br>0.7631
S2	B	**Wild-type Vs. *Eif4e*$^{Ki/Ki}$**<br>30 min= 198.6 Vs 114.1<br>120 min= 194.2 Vs 108.6<br>180 min= 223.0 Vs 96.0	Wild-type= 6<br>*Eif4e*$^{Ki/Ki}$ = 6	Student t-test.		**Wild-type Vs. *Eif4e*$^{Ki/Ki}$**<br>30 min= 0.062<br>120 min= 0.091<br>180 min= 0.063

S3	A	**Wild-type Vs. *Eif4e$^{Ki/Ki}$***<br>eIF4G = 0.97 Vs 1.32<br>CYFIP1= 0.78 Vs 1.2<br>FMRP= 1.43 Vs 0.82<br>T-eIF4E= 1.25 Vs 1.7<br>T-BP2= 1.55 Vs 2.1<br>Arc = 0.94 Vs 0.56	eIF4G = 10<br>CYFIP1= 12<br>FMRP = 6<br>T-eIF4E =13<br>T-BP2 = 12<br>Arc = 13	Multiple t-test.	Holm-Sidak Method	**Wild-type Vs. *Eif4e$^{Ki/Ki}$***<br>eIF4G = 0.3234<br>CYFIP1= 0.3361<br>FMRP= 0.2139<br>T-eIF4E= 0.2319<br>T-BP2= 0.2537<br>Arc= 0.3545
	B	**Wild-typeVs. *Eif4e$^{Ki/Ki}$***<br>eIF4G = 26.0 Vs -9.1<br>CYFIP1= -14.6 Vs 27.9<br>FMRP= -24.3 Vs 8.5<br>T-eIF4E= 25.0 Vs 20.6<br>P-BP2= 39.6 Vs 22.0<br>T-BP2= -11.9 Vs 4.6<br>Arc = 71.0 Vs 31.0	eIF4G = 12<br>CYFIP1= 14<br>FMRP= 8<br>T-eIF4E=16<br>P-BP2= 6<br>T-BP2= 11<br>Arc= 17	Multiple t-test.	Holm-Sidak Method	**Wild-type Vs. *Eif4e$^{Ki/Ki}$***<br>eIF4G = 0.02234<br>CYFIP1= 0.00007<br>FMRP= 0.0072<br>T-eIF4E= 0.7712<br>P-BP2= 0.0810<br>T-BP2= 0.0756<br>Arc= 0.018
S7	A	**Wild-type (Up vs Down)**<br>5´UTR length – 218.5 vs 160.0<br>5´UTR GC content – 65.96 vs 57.95<br>5´UTR Gibbs free energy – -85.10 vs -55.95	N= 404 vs 55	Mann-Whitney test		**Wild-type**<br>5´UTR length = 0.0132<br>5´UTR GC content = 0.0001<br>5´UTR Gibbs free energy = 0.0003
	B	***Eif4e$^{Ki/Ki}$* (Up vs Down)**<br>5´UTR length – 200.5 vs 176.0<br>5´UTR GC content - 60.99 vs 64.86<br>5´UTR Gibbs free energy – -69.15 vs -74.80	N= 326 vs 89			***Eif4e$^{Ki/Ki}$***<br>5´UTR length = 0.2170<br>5´UTR GC content = 0.0020<br>5´UTR Gibbs free energy = 0.9569
S8	A	**Wild-type Vs. *Eif4e$^{Ki/Ki}$***<br>Dvl2 = 1.24 Vs 1.1<br>Fzd4 = 1.10 Vs 1.13<br>Sfrp1= 0.15 Vs 0.19	Dvl2 = 6<br>Fzd4= 6<br>Sfrp1 = 6	Multiple t-test.	Holm-Sidak Method	**Wild-type Vs. *Eif4e$^{Ki/Ki}$***<br>Dvl2 = 0.5651<br>Fzd4= 0.8947<br>Sfrp1= 0.5171
	B	**Wild-type Vs. *Eif4e$^{Ki/Ki}$***<br>Dvl2 = 70.1 Vs 13.8<br>Fzd4 = 3.44 Vs 4.9<br>Sfrp1= -5.50 Vs 8.87	Dvl2 = 6<br>Fzd4= 5<br>Sfrp1 = 5	Multiple t-test.	Holm-Sidak Method	**Wild-type Vs. *Eif4e$^{Ki/Ki}$***<br>Dvl2 = 0.0295<br>Fzd4= 0.8320<br>Sfrp1= 0.0142

SUPPLEMENTAL INFORMATION

Supplemental Figure Legends

Figure S1. Input-output curves of medial perforant path-DG evoked responses show normal basal synaptic transmission and excitability in *Eif4e*$^{ki/ki}$ mice. Related to Figure 1.

(A) Input-output relationship of the fEPSP slope in *Eif4e*$^{ki/ki}$ and wild-type littermates. Current intensities ranged from 80-300 µA. No significant difference between groups (n = 4, p = 0.9974; Two-way ANOVA).

(B) Population spike (Spike) amplitude (p = 0.5977).

(C) EPSP-Spike plot. p= 0.7631). Values are mean ± SEM.

Related to

Figure S2. Population spike LTP in *Eif4e*$^{ki/ki}$ mice. Related to Figure 1.

(A) Time-course plots of medial perforant path-DG evoked population spike amplitude recorded before and after high-frequency stimulation (HFS, indicated by arrow) in homozygous *Eif4e*^{S209A} knockin mice (*Eif4e*$^{ki/ki}$; n=7), heterozygous mice (*Eif4e*$^{+/ki}$; n=8) and wild-type littermates (*Eif4e*$^{+/+}$ mice; n=10). Values are mean (± SEM) expressed in percent of baseline.

(B) Bar graphs of mean changes in population spike amplitude recorded between 0-10 min, 30-40 min, and 170-180 min post-HFS. There was no significant difference between genotypes at these time points. Student's t-test.

Figure 1C shows representative field potentials.

Figure S3. Basal and post-HFS expression of translation factors in DG lysates. Related to Figure 2.

(A) Immunoblot analysis eIF4G, eIF4E, 4E-BP2, CYFIP1, FMRP and Arc in DG lysates from naïve mice (basal state). Expression is normalized GAPDH. No significant difference between wild-type and *Eif4e*$^{ki/ki}$ mice. Values are means + SEM.

(B) Immunoblot analysis of eIF4G (n=12, 0.02234), eIF4E (n=16, 0.7712), 4E-BP2 (n=11, 0.0756), CYFIP1 (n=14, 0.00007), FMRP (n=8, 0.0072) and Arc (n=17, 0.018) in HFS-treated DG. Values are mean (± SEM) expressed as percent change relative to contralateral DG. Significant differences between genotypes are indicated. (*$p < 0.05$, **$p < 0.001$, ***$p < 0.0001$, Multiple t-test).

(C) Representative immunoblots for panel (B).

Figure S4. Quality Control (QC) of ribosome profiling experiment. Related to Figure 3.

(A) Graphical depiction of groups presented in this figure; LFS: low frequency stimulation, HFS: high frequency stimulation; in *Eif4e*$^{ki/ki}$ and *Eif4e*$^{+/+}$ (wild-type) mice ribosome profiling experiment in Fig. 3.

(B) (Top panel) Size of footprints and total mRNA libraries analysed; frequency of different size fragments (length of mapped reads; bp). (Bottom) Fraction of reads within start codon window for frames 1, 2 and 3.

(C) Periodicity of reads (frequency) around the start and stop codons (nt from) for the ribosome profiling experiment (footprints & total mRNA) for the experimental groups shown (see legend for A.).

Figure S5. Comparison of transcription, translation and gene ontology in HFS vs contralateral control and test-pulse LFS control in *Eif4e*$^{+/+}$ (wild-type) mice. Related to Figure 3.

A. Scatter plot of TE or RPKM comparisons for the indicated groups: low-frequency test-pulse stimulation (LFS); standard LTP protocol of test-pulse stimulation and high-frequency stimulation (HFS); in *Eif4e*$^{ki/ki}$ and *Eif4e*$^{+/+}$ (wild-type) mice. HFS-treated ipsilateral DG is compared to contralateral, non-stimulated DG and to ipsilateral DG of mice given LFS only.

B. Gene Ontology analysis using IPA canonical pathways for DTGs and DEG. Top 3 groups are shown.

Figure S6. Gene ontology analysis for upregulated DTGs in *Eif4e*$^{ki/ki}$ mice. Related to Figure 3.

Gene ontology analysis of downregulated genes in *Eif4e*$^{+/+}$ mice (57 genes) and *Eif4e*$^{ki/ki}$ mice (91 genes); plots for biological process (A) cellular component (B) and molecular function (C) with number of genes in each category with p-values. (D) KEGG pathway analysis for upregulated genes. Mean ± SEM and Student's t-test in **Table S1**.

Figure S7. 5'-UTR sequence analysis using UTRscan. Related to Figure 3.

(A) (TOP) Summary table depicting differences in the variables (length, %GC and Gibbs Free energy) shown in bar graphs. (BOTTOM) Length (nt; nucleotides), %GC content and Gibbs free energy (kcal/mol) are shown for upregulated and downregulated DTGs from ribosome profiling experiment (WT 40 min HFS and KI 40 min HFS).

(B) (TOP) Summary table depicting differences in motifs (uORF, TOP, IRES, PG4) shown in bar graphs. (BOTTOM) Bar graphs for UTRscan motif analysis in for upregulated and downregulated DTGs from ribosome profiling experiment (WT 40 min HFS and KI 40 min HFS). Data are shown as mean ± SEM. For A; Mann-Whitney test ***$p<0.001$, *$p<0.05$.

Figure S8. Immunoblot analysis validates phospho-eIF4E-dependent expression of Wnt pathway receptor, dishevelled 2 (Dvl2), following LTP induction in the dentate gyrus. Related to Figure 4.

A) Immunoblot analysis of Dvl2, Fzd4, and Sfrp1 in DG lysates from naïve mice (basal state). Densitometric values are normalized to GAPDH. Values are means + SEM. No significant difference between wild-type and *Eif4e*$^{ki/ki}$ mice.

(B) Changes in the expression of Dvl2, Fzd4, and Sfrp1 in DG lysate following HFS. Values are expressed in percent change relative to contralateral DG control. Gives stats for genotype comparison.

(C) Representative immunoblots for panel (B). HFS = high-frequency stimulation. (+) Ipsilateral DG, (-) Contralateral DG.

Figure S9. Comparison of ribosome profiling DTGs in present study **with previous studies. Related to Figure 3.** Venn diagrams showing comparison of ribosome profiling DTGs at baseline or 40 min post-HFS (*Eif4e*$^{+/+}$ vs *Eif4e*$^{ki/ki}$) from the present study with (**A)** 842 FMRP HITS-CLIP targets[1] and (**B)** 91Mnk1 targets identified by BONCAT and SILAC labeling in BDNF-treated cultured cortical neurons[2].

Supplemental References

[S1]. Darnell, J.C., Van Driesche, S.J., Zhang, C., Hung, K.Y., Mele, A., Fraser, C.E., Stone, E.F., Chen, C., Fak, J.J., Chi, S.W., et al. (2011). FMRP stalls ribosomal translocation on mRNAs linked to synaptic function and autism. Cell. *146*, 247–261.

[S2]. Genheden, M., Kenney, J.W., Johnston, X.H.E., Manousopoulou, A., Garbis, S.D., and Proud, X.C.G. (2015). BDNF stimulation of protein synthesis in cortical neurons requires the MAP kinase-interacting kinase MNK1. J Neurosci *35*, 972–984. 10.1523/JNEUROSCI.2641-14.2015.
